# Anisotropic, Wrinkled, and Crack-Bridging Structure for Ultrasensitive, Highly Selective Multidirectional Strain Sensors

**DOI:** 10.1007/s40820-021-00615-5

**Published:** 2021-05-04

**Authors:** Heng Zhang, Dan Liu, Jeng-Hun Lee, Haomin Chen, Eunyoung Kim, Xi Shen, Qingbin Zheng, Jinglei Yang, Jang-Kyo Kim

**Affiliations:** 1 Department of Mechanical and Aerospace Engineering, The Hong Kong University of Science and Technology, Clear Water Bay, Kowloon, Hong Kong, People’s Republic of China; 2 School of Science and Engineering, The Chinese University of Hong Kong, Shenzhen, 518172 Guangdong People’s Republic of China

**Keywords:** Anisotropic strain sensor, Wrinkle, Aligned carbon nanotube, Stretchability, Complex motion detection

## Abstract

Flexible multidirectional strain sensors are crucial to accurately determining the complex strain states involved in emerging sensing applications. Although considerable efforts have been made to construct anisotropic structures for improved selective sensing capabilities, existing anisotropic sensors suffer from a trade-off between high sensitivity and high stretchability with acceptable linearity. Here, an ultrasensitive, highly selective multidirectional sensor is developed by rational design of functionally different anisotropic layers. The bilayer sensor consists of an aligned carbon nanotube (CNT) array assembled on top of a periodically wrinkled and cracked CNT–graphene oxide film. The transversely aligned CNT layer bridge the underlying longitudinal microcracks to effectively discourage their propagation even when highly stretched, leading to superior sensitivity with a gauge factor of 287.6 across a broad linear working range of up to 100% strain. The wrinkles generated through a pre-straining/releasing routine in the direction transverse to CNT alignment is responsible for exceptional selectivity of 6.3, to the benefit of accurate detection of loading directions by the multidirectional sensor. This work proposes a unique approach to leveraging the inherent merits of two cross-influential anisotropic structures to resolve the trade-off among sensitivity, selectivity, and stretchability, demonstrating promising applications in full-range, multi-axis human motion detection for wearable electronics and smart robotics.

**Supplementary Information:**

The online version contains supplementary material available at 10.1007/s40820-021-00615-5.

## Highlights

- Two functionally different anisotropic layers are rationally assembled for highly selective and stretchable multidirectional strain sensors.
- Concurrently excellent selectivity, sensitivity, stretchability, and linearity up to 100% strain is demonstrated for the first time in a multidirectional strain sensor.
- A novel stepwise crack propagation mechanism is proposed to enable high stretchability and linearity.

**Supplementary Information:**

The online version contains supplementary material available at 10.1007/s40820-021-00615-5.

## Graphical abstract

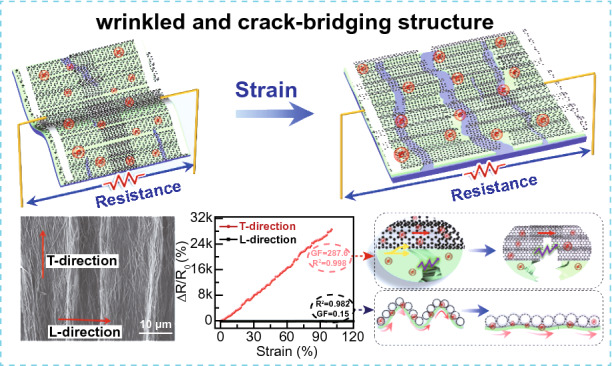

## Introduction

Highly stretchable strain sensors, as indispensable components of wearable electronics, have attracted extensive interests in the fields of intelligent robotics [1, 2], human–machine interface [3–7], human motion detection [8–11], and personal health-care monitoring system [12–15]. Much effort has been made to precisely convert mechanical stimuli to digital signals by improving the stretchability, sensitivity, linearity, and durability of stretchable sensors [16]. Resistive-type sensors made by depositing conductive filler networks on flexible and stretchable polymer substrates are promising contenders because of the synergy arising from the conductive nanofillers and stretchable polymer matrices. However, there is always a trade-off between superior stretchability and high sensitivity because of conflicting structural requirements based on different principles [17–21]. The high sensitivity stems from the interruption of conductive paths under a tiny change in strain, while the excellent stretchability requires the sensor to retain the conductive paths even under large deformations [22]. Several attempts have been made toward combining high stretchability with high sensitivity by integrating a sliding mechanism with the cracked structures. For example, a layer-structured, hierarchical conductive network based on 2D MXene sheets and 1D Ag nanowires (AgNWs) allowed sliding between the layers so as to control crack propagation under strain, delivering a large gauge factor (GF) of up to 8700 and stretchability of over 80% [23]. The GF is a measure of the sensitivity of strain sensors and defined as the relative resistance change over applied strain. Other elaborate structures, such as 0D fullerene-1D silver nanowire (AgNW)-2D graphene oxide (GO) ternary nanocomposites [24], conductive coaxial structures [18], multilayer AgNW–MXene/WPU fibers [19], nanoparticle–nanosheet hybrid network [20], carbonized plain woven cotton fabrics [25], and overlapped carbon nanotubes (CNTs) [26], were also prepared to achieve GFs higher than 100 and stretchability over 70% by taking advantage of the slippage between the adjacent conductive fillers and controlling the percolation networks. Nonetheless, most of these existing highly stretchable and sensitive strain sensors were only capable of detecting strain amplitudes without distinguishing the loading directions, while experiencing poor linearity. A highly linear response throughout the working range avoids the elaborate calibration while directional sensing allows the detection of complex strain states, both of which are essential to extensive applications of stretchable strain sensors.

Flexible strain sensors with anisotropic electromechanical behaviors are highly desired for detecting complex multidimensional strains encountered in practical applications. The anisotropic sensing capability was realized by utilizing a structure having both wrinkles and cracks [27–29]. An extremely high sensitivity with a GF over 1000 was achieved in the crack opening direction for the crack-size tuned, wrinkled gold E-skin while being insensitive (GF < 1) in the direction transverse to it. The anisotropic sensing behavior was translated to high selectivity of 30.3, a critical parameter required for a directional sensing capability [30–32]. However, these crack-based sensors suffered from very limited stretchability of less than 20% [33, 34]. An alternative strategy is to introduce aligned structures as conductive components, such as aligned CNTs or CNT/graphene hybrids [21, 35–40], aligned carbon nanofibers (CNFs) [30], vertically aligned graphene [41], and oriented carbonized cellulose fibers [42]. These sensors featured immense stretchability (> 200%) in the alignment direction, while the linearity and sensitivity were relatively low with GFs usually less than 100. Such low GFs arising from the sliding between the aligned conducting fillers without creating cracks also resulted in low selectivity of less than 2 at strains below 5%, inadequate to differentiate the loading directions [35, 37, 42–44]. These limitations impede their applications in wearable electronics and artificial skins which require accurate tracking of multiaxial strains with wide sensing ranges over 100% strain [26, 45]. It is challenging to integrate the mutually exclusive properties, such as high sensitivity, high selectivity, and broad working range, i.e., high stretchability in all directions, with consistent linearity in a sensor using an either anisotropic structure alone.

With the above backdrop in mind, we propose here a synergistic strategy of combining two anisotropic structures, namely periodically wrinkled and microcracked CNT–GO hybrid films and aligned CNT arrays, to leverage their inherent merits of high sensitivity of cracked films and high stretchability of aligned CNTs. These two functionally different anisotropic layers were rationally assembled on an elastomer substrate to form a bilayer composite sensor. The top-aligned CNT layer was aimed to bridge the microcracks in the underlying CNT–GO film giving rise to both ultrahigh GFs and excellent stretchability, while the periodic wrinkles were insensitive to the transverse strains giving rise to high selectivity. The ultrasensitive, highly selective multidirectional strain sensor completed with excellent stretchability and linearity may find diverse wearable applications requiring accurate detection of full-range, multi-degree-of-freedom human motions.

## Experimental Section

### Materials and Methods

#### Materials

High purity ($>$ 95%) single-walled CNTs were supplied by Chengdu Organic Chemical Co. Ltd. The surfactant Triton X-100, dopamine, tris(hydroxymethyl)aminoethane, and hydrochloride were supplied by Sigma-Aldrich. Deionized water was used throughout the work. Dopamine (0.02 g mL^−1^) was dissolved in 10 mM Tris–HCl (pH 8.5) [46].

#### Fabrication of Anisotropic Strain Sensor

The CNT dispersion was prepared by dispersing 5 mg CNTs in 100 mL deionized water with the aid of 1 g Triton X-100 using a probe sonication (Sonic VCX 750) at 400 W for 30 min [47]. The GO dispersion of 0.5 mg mL^−1^ was prepared from graphite precursor following the modified Hummers method [48]. The CNT–GO hybrid film was obtained by vacuum infiltration of the mixed solution of CNT and GO on a cellulose membrane (pore size 0.22 μm, N8645-100EA, SIGMA), as shown in Fig. S1a. Subsequently, the CNT–GO film was transferred to a pre-strained (150%) elastic tape (VHB4910, 3 M, Inc) and washed by acetone to remove the cellulose membrane. After releasing the pre-strain, the substrate gradually returned to its original state, forming wrinkles and microcracks in the film.

**Fig. 1:**
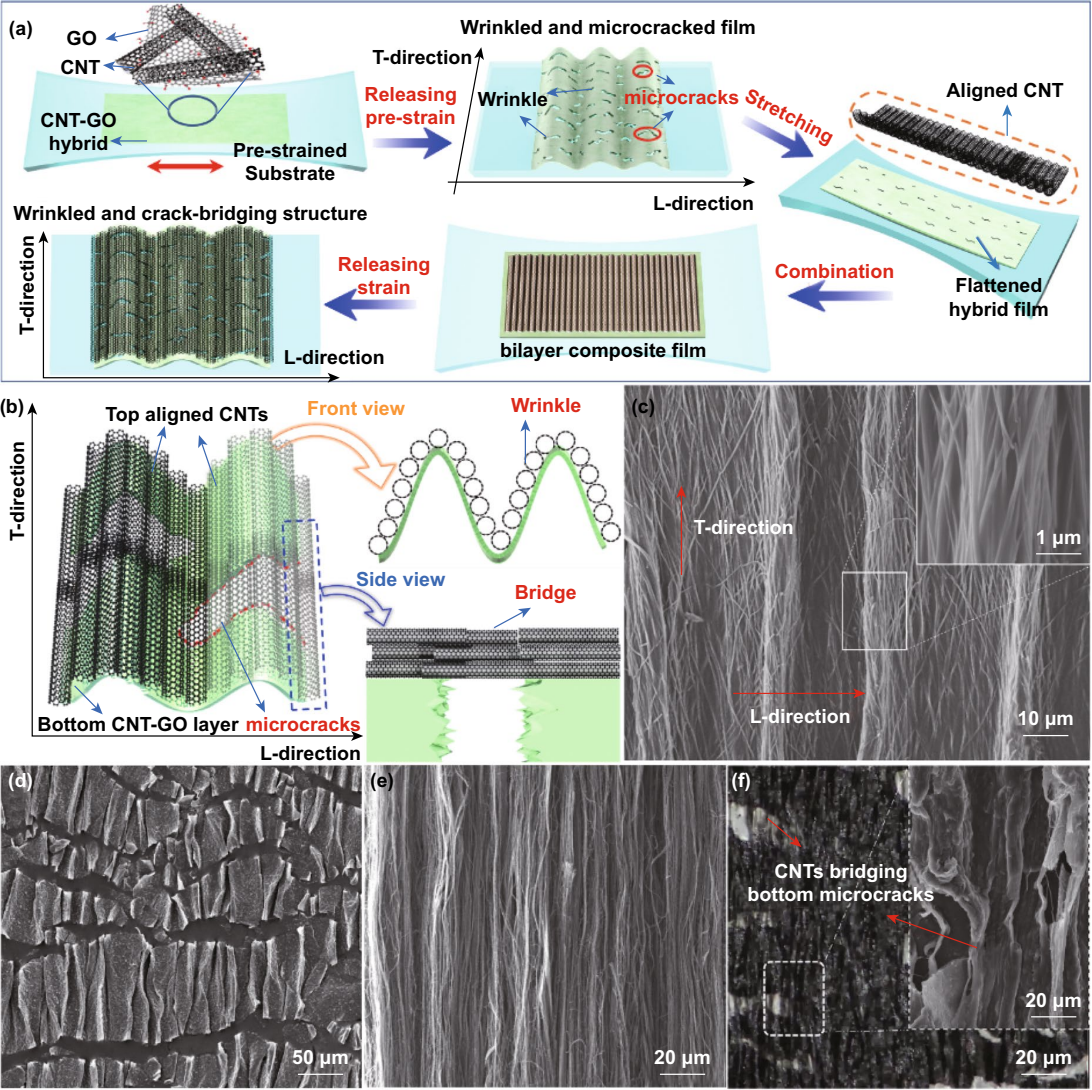
Flexible anisotropic strain sensor made of rationally assembled bilayers of periodically wrinkled and cracked CNT–GO hybrid film and aligned CNT arrays. **a** Schematic illustration of the fabrication process. **b** Schematic illustration of the bilayers of wrinkled and crack-bridging structures. **c** SEM images of bilayers showing the CNTs aligned in T-direction with underlying wrinkles in L-direction: wrinkle ridge in inset. **d** SEM image of CNT–GO film showing both extensive wrinkles and microcracks. **e** SEM image of aligned CNT arrays. **f** Optical and SEM images of bilayer film showing the bottom microcracks bridged by the top-aligned CNTs

Vertically aligned CNT arrays of 500 μm in height were grown on silicon wafers using chemical vapor deposition [49]. To fabricate aligned CNTs film, a piece of CNT arrays was placed on a cellulose membrane and laid down by applying a shear force using a blade, forming a horizontally aligned CNT film (Fig. S1b). The mixture of octane and mineral (2:1, v:v) was used as lubricant during this process. The obtained aligned CNT film was cut into a rectangle, followed by rinsing with octane and immersing in a polydopamine (PDA) solution while magnetic stirring for 24 h. A mild stirring speed of 400 rpm was used to avoid damage to the aligned structure (Fig. S2). The collected aligned CNT–PDA was bonded to the flattened CNT–GO film in the orthogonal direction (Fig. 1a). After removing the membrane by acetone and releasing the applied strain, the final anisotropic wrinkled and crack-bridged structure was obtained. To optimize the structural parameters, aligned CNT–PDA films with five different areas, namely 5 × 10, 10 × 10, 15 × 10, 17 × 10, and 20 × 10 mm^2^, were prepared. All films were cut into equal sizes and evenly distributed on the bottom layer with a GO content fixed at 25%. Four different CNT–GO hybrid films with different CNT to GO ratios between 0 to 75% were prepared. After assembling aligned CNTs on top of the CNT–GO film to form a bilayer sensor, copper wires were connected to two edges of the sensor using silver paste (Fig. S3), and another layer of elastic tape was applied to fully encapsulate the sensor. For testing anisotropic sensing performance, copper wires were connected to two edges of the bilayer film along either L- and T-direction depending on the direction of applied strain so that the resistance could be measured in the same direction. Two bilayer films with copper wires connected in their respective T-directions were orthogonally stacked to prepare multidirectional sensors with the CNT alignment being perpendicular to each other (Fig. S4).

#### Fabrication of Human Motion Capture Device

The sensors mounted on human body were connected to a commercial circuit board (Arduino UNO). The motion-induced resistance changes were converted into digital data by the signal processing circuit. The motion recognition and amination were performed on a computer using Unity software according the data received.

### Characterization

The morphologies of sensors were characterized using a field-emission scanning electron microscopy (SEM, JSM-7100). An optical microscope (Olympus LC30) was used to characterize the morphological variations during the loading/unloading cycles. The X-ray photoelectron spectroscopy (XPS, PHI5600, Physical Electronics) was used to quantitatively measure the chemical compositions of CNTs with and without PDA treatment. The electrical conductivity was measured using a four-point probe method (Ecopia HMS-5500). The electromechanical performance of sensors was evaluated on a universal testing machine (MTS I2) where a digital multimeter (34970A Data Acquisition/Data Logger Switch Unit, Agilent) was used to continuously record the corresponding resistance changes with strains.

### Molecular Dynamics (MD) Simulations

MD simulations were carried out using the software Materials Studio. The COMPASS forcefield was used for describing all the atomic interactions. The interaction energies between CNTs, CNTs and GO, and top-aligned CNT and bottom CNT–GO films were calculated (see Supporting Information for details). The system consisted of three representative elements including single-walled CNTs, GO sheets and PDA (Fig. S16) [50–52]. The atom-based method with a cutoff of 15.5 Å and Ewald summation with sum tolerance of ${10}^{-4}$ kcal mol^−1^ were used to describe the van der Waals and long-range Coulomb interactions [53].

## Results and Discussion

### Fabrication and Structural Features of the Anisotropic Strain Sensor

The fabrication procedure of the anisotropic bilayer structure is schematically shown in Fig. 1a (see Experimental Section and Fig. S1, Supporting Information for details). The sensor consisted of two layers, namely the bottom CNT–GO hybrid film and the top-aligned CNT array. The CNT–GO film prepared by vacuum filtration of filler dispersion (Fig. S1a) was transferred to a uniaxially pre-strained elastomer tape (VHB4910, 3 M, Inc). The excellent stretchability of VHB substrate ensured an ultrahigh failure strain of over 500% at room temperature (Fig. S5), making it possible to design composite sensors with an ultrawide working range. A large pre-strain of 150% was applied in the longitudinal direction (designated as “L-direction” hereafter) to achieve extensive cracks once the pre-strain was released by exploiting the expansion of elastomer substrate in the transverse direction (T-direction) because of the Poisson’s effect. The strong adhesion between the elastomer substrate and the CNT–GO film led to the out-of-plane buckling of the film upon release of pre-strain (Fig. S6a-d)), forming densely distributed periodic wrinkles in the L-direction with their ridges aligned in the T-direction. The adhesion between the wrinkled CNT–GO film and substrate remained strong because of the highly adhering elastomer tape (Fig. S6e-g). Subsequently, the polydopamine-coated aligned CNT array (CNT–PDA, Fig. S1b) was placed on top of the uniaxially pre-strained hybrid film, with the CNT alignment in the T-direction. After pressing the assembly and releasing the pre-strain, an anisotropic, wrinkled, and crack-bridging bilayer structure was obtained with periodic wrinkles in the L-direction and aligned CNTs bridging the microcracks in T-direction, as shown in Fig. 1b, c. The bottom CNT–GO film featured orthogonally arranged periodic wrinkles and microcracks, as shown in Fig. 1d. These wrinkles were aimed to reduce the sensitivity of sensor in the L-direction, while parallel microcracks were essential to achieving superior sensitivity in the T-direction. The orthogonal layout was designed to yield high selectivity of the sensor. Meanwhile, the top CNT film with a high degree of alignment (Fig. 1e) effectively bridged the microcracks in the bottom layer (Fig. 1f), which was necessary to achieve high stretchability in the T-direction. Therefore, the assembled anisotropic structure consisting of periodic wrinkles in the L-direction and crack-bridging, aligned CNTs in the T-direction was expected to deliver a highly selective sensing capability as well as exceptional sensitivity and stretchability when loaded in the T-direction.

### Optimization of Sensing Performance in the T-direction

**Fig. 2:**
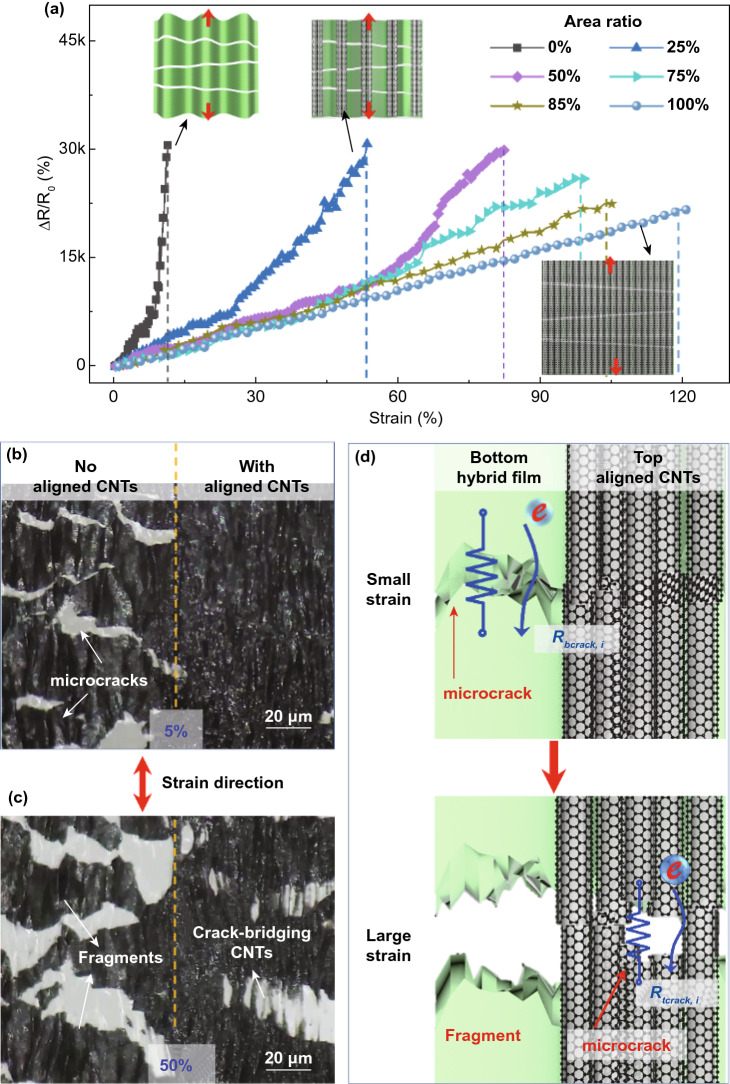
Optimization of stretchability and linearity of the sensor. **a** Relative resistance changes in T-direction of bilayer sensors with different area ratios of top to bottom films: schematics of the corresponding structures in inset. Morphologies of bilayer sensors when loaded at **b** 5% and **c** 50% strains in T-direction. **d** Schematic illustration of stepwise crack propagation mechanism

The key performance of the bilayer sensor in the T-direction in terms of sensitivity, stretchability, linearity, and long-term stability was determined by several material and structural parameters relating to individual layers and their interlayer bonding. The amount of CNTs used in the top-aligned CNT film was first optimized. More CNTs in the top film meant a higher area ratio, defined as the ratio of the area of top-aligned CNTs to the area of bottom CNT–GO layer. The electromechanical performance was investigated by measuring the normalized resistance change, ∆*R*/*R*_0_ where *R*_0_ is the initial resistance and ∆*R* is the change in resistance, under tension. The resistance change in the L-direction was negligible regardless of area ratio (Fig. S7), while that in the T-direction showed large variations depending on the area ratio (Fig. 2a). When the area ratio was 0, namely without top CNT film, the bottom CNT–GO film was extremely sensitive with a GF $\approx$ 3000 because of the microcracks, but the stretchability was noticeably low with less than 10% in the T-direction. The stretchability consistently increased at the expense of reduced sensitivity with increasing area ratio. For human motion detection, simultaneously high stretchability of over 100% and a high GF of more than 100 are desired [54]. Therefore, an optimal area ratio of 85% was required to deliver balanced stretchability of 110% and a GF of 220. After the introducing the top-aligned CNT layers, the crack propagation in the bottom film was alleviated because of the crack-bridging by aligned CNTs, contributing to reduced sensitivities compared to the neat CNT–GO film at the same strain. The sensors with area ratios lower than 85% possessed a bilinear response, while those with area ratios of 85% or higher showed good linearity in the whole working range.

To explain the high stretchability in the T-direction, a stepwise crack propagation mechanism enabled by the top CNT film was revealed, as shown in Fig. 2b–d (full information in Figs. S8-S11 and Video S1). Under a small tensile strain of 5% in T-direction, microcracks were initiated in the bottom layer while the top layer remained intact (Fig. 2b). The crack opening in the bottom layer prompted a drastic increase in tunneling resistance (${R}_{b\mathrm{crack},i}$), which was responsible for the changes in resistance and GF at low strains (Fig. 2d, top panel). When the strain was increased to 50% and beyond, the gaps between the fragments in the bottom layer further widened, while cracks were finally propagated in the top CNT film (Fig. 2c), which augmented the tunneling resistance between the aligned CNTs (${R}_{t\mathrm{crack},i}$) and progressively dominated the whole resistance change of the sensor (Fig. 2d, bottom panel). Nevertheless, the widened gaps between the fragments were bridged by bundles of aligned CNTs in the top layer, preventing the rupture of conductive networks at high strains and thus maintaining high stretchability. As the area ratio was increased, more CNTs were made available to bridge the microcracks even at large strains, giving rise to enhanced stretchability.

To probe the source of excellent linearity at high area ratios of over 85%, the above stepwise crack propagation mechanism is quantitatively expressed in Eqs. S5 and S8 for small and large strains, respectively, with reference to the equivalent circuit models given in Figs. S10 and S11. For conventional crack-based sensors, an exponential increase in resistance at large strains was commonly observed because of the rapid crack propagation [30]. The stepwise crack propagation in our bilayer sensor, in contrast, slowed the crack propagation at large strains through increasing the area ratio to over 85%, making the ∆*R*/*R*_0_ plot straight at large strains as indicated by the fitting of experimental results in Fig. S12 and Table S2. In other words, an almost linear ∆*R*/*R*_0_ response over the whole working range was made possible by the crack-bridging action of the CNTs in the top layer which moderated the crack propagation at large strains. Such extraordinary linearity across the whole 100% strain is unprecedented for stretchable sensors, which is essential to detection of wide-range human motions [55].

**Fig. 3:**
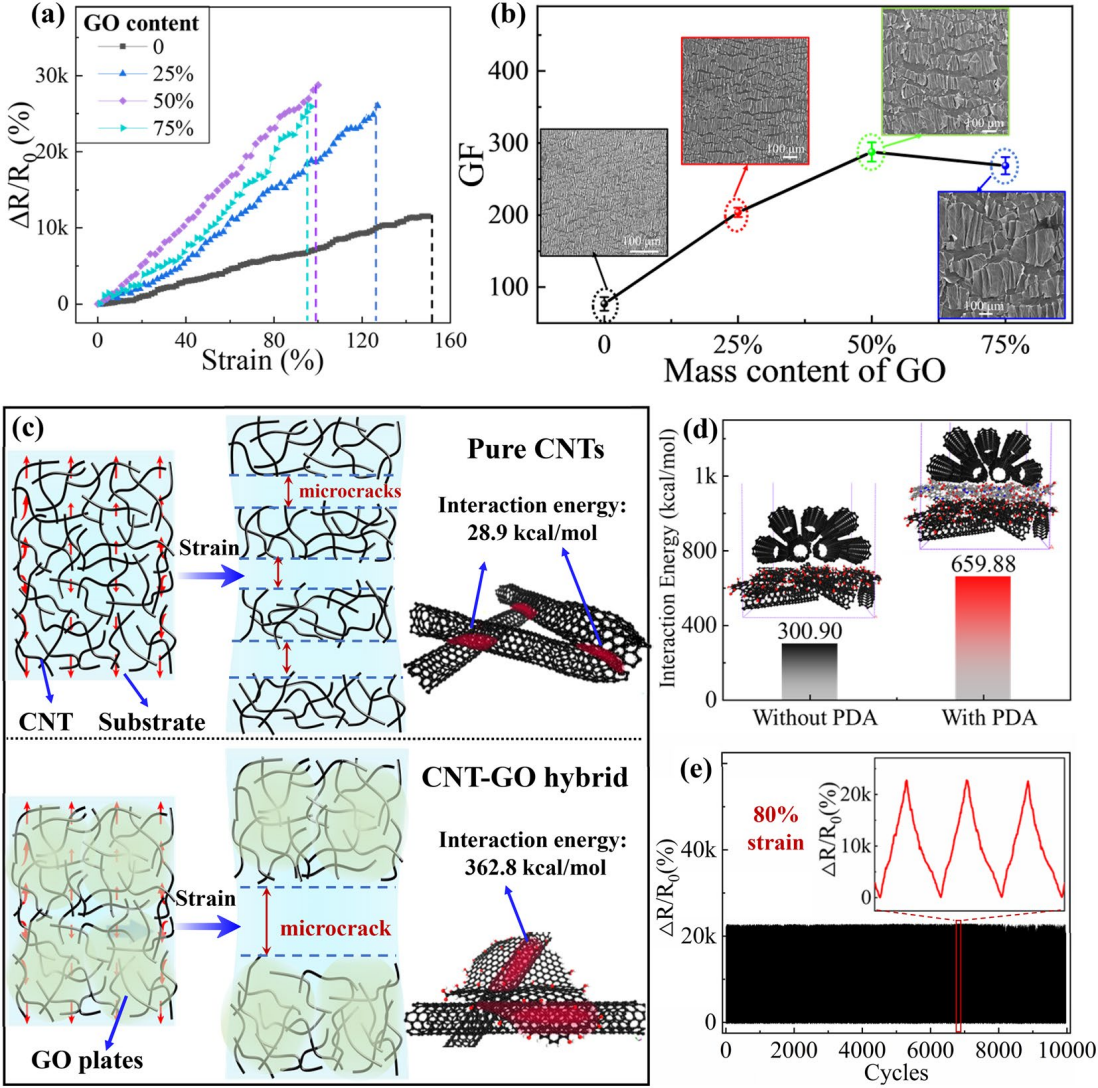
Optimization of sensitivity and cyclic stability of the sensor. **a** Relative resistance changes in T-direction of bilayer sensors with different GO contents in the bottom CNT–GO film. **b** GFs of sensors prepared with different GO contents: SEM images of the bottom layers at 2% strain in inset. **c** Mechanism of morphology change after adding GO sheets. **d** Interaction energies between the top and bottom layers. **e** Resistance changes for up to 10,000 loading/unloading cycles at 80% strain showing excellent durability and stability of the sensor

The addition of top CNT layer improved the stretchability of the sensor but inevitably sacrificed the GF. To remedy the sensitivity, the number of microcracks in the bottom GO-CNT film was tuned by varying the GO mass content (Fig. 3a). The increasing GO content gave rise to a smaller number of microcracks (insets in Fig. 3b), leading to larger gaps between the adjacent fragments than in the pure CNT film when stretched to the same strain (Fig. 3c). Consequently, the GF in the T-direction rose first to around 280 at a GO content of 50% (Fig. 3b). The fewer microcracks with more GO was attributed to the enhanced interaction energy according to the MD simulations (Fig. 3c). The contact between a 1D CNT and a 2D GO sheet in the CNT–GO film occurred by lines, giving rise to an order of magnitude higher interaction energy than between 1D CNTs in the pure CNT film where point contacts dominated. Apart from the larger contact area, the amphiphilic nature of GO sheets also contributed much to the enhanced energy [56, 57]. The higher interaction energy meant the hybrid film being stronger than the pure CNT counterpart, leading to fewer microcracks when more GO sheets were added in the bottom film. However, an excessive amount of GO sheets in the hybrid film tended to increase the initial resistance, and thus reduced GF at a GO content of 75% (Fig. S13). It is also noted that the larger number of microcracks created in the pre-cracking step contributed to the high stretchability of 100% and excellent linearity of the sensor, which could not be achieved in the sensor fabricated without the pre-cracking process (Fig. S14).

In addition to the structural parameters of the two layers, the strong adhesion between them is also critical to the long-term performance of sensors under repeated loading cycles. The top CNT films were treated with PDA to improve the adhesion with the bottom hybrid film (Fig. S15). The presence of benzene rings and polar amino/hydroxyl groups in PDA imparted stronger interaction between the two layers through π-π interactions and hydrogen bonds, leading to more than doubled interaction energy than that without treatment (Figs. 3d and S16). The CNT alignment in the film with treatment remained intact after repeated loading/unloading cycles (Fig. S17a) because of the strong adhesion, positively contributing to the stable and consistent resistance changes of the sensor under cyclic loading (Fig. 3e). On the contrary, the CNTs without treatment became highly disordered after cycles (Fig. S17b) due probably to serious interlayer sliding, causing severely fluctuated resistance changes (Fig. S18). In addition, the response time of the sensor was slightly increased from 489 to 538 ms even after 10,000 cycles of tension (Fig. S19), demonstrating its exceptional durability for long-term use. Compared to PDMS-based strain sensors with a typical response time of 130 ms [20, 42], the longer response time of our sensor was attributed to the more viscous nature of VHB than PDMS (Fig. S20).

Overall, the multifold merits of the bilayer sensor in the T-direction were achieved through optimizing a few important structural parameters. The top-aligned CNT film enabled the stepwise crack propagation, underpinning the excellent stretchability and linearity. The sensitivity was improved by adding GO sheets in the bottom hybrid film so as to enhance the interactions between the 1D and 2D fillers, yielding fewer but wider microcracks and thus a larger change in the resistance than the pure CNT film when loaded. The enhanced adhesion between the two layers by PDA treatment guaranteed long-term stability under cyclic loadings.

### Anisotropic Electromechanical Performance

**Fig. 4:**
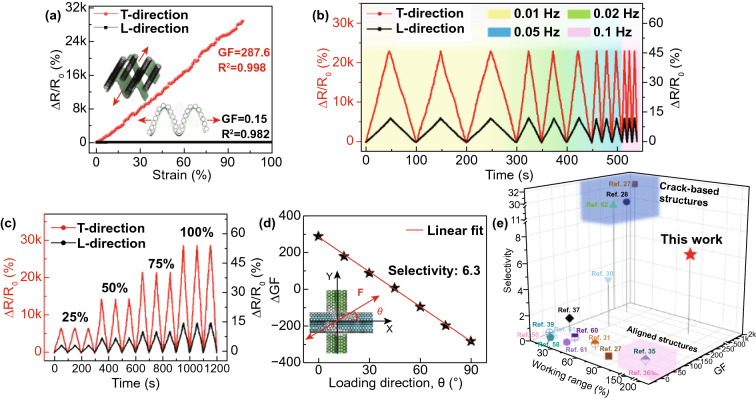
Anisotropic sensing performance of the sensor. **a** Relative resistance changes as a function of strain in L- and T-directions. **b** Relative resistance changes under 80% strain at different frequencies. **c** Relative resistance changes under repeated cycles at different strains. **d** Selectivity calculated from the linear fit of ΔGF versus loading direction data. **e** Comparison of GF, selectivity, and working range with existing anisotropic strain sensors in the literature. The highlighted blue and pink regions represent sensors with crack-based and aligned structures, respectively

In addition to the aforementioned multifold merits in the T-direction, the sensor with an optimal area ratio of 85% and a GO content of 50% showed highly anisotropic electromechanical responses to tensions applied in the orthogonal directions, as shown in Fig. 4a. When loaded along the L-direction, ∆*R*/*R*_0_ remained almost unchanged with a low GF of only 0.15. The tensile strain applied in the L-direction expended only to flatten the wrinkles without altering the conductive networks of the entire sensor (Figs. S21–S23 and Video S2), causing negligible resistance changes. By contrast, ∆*R*/*R*_0_ increased linearly (*R*-squared greater than 98%) as the strain rose to 100% with an ultrahigh GF of 287.6 when the tension was applied in the T-direction. The linear working range of our bilayer sensor is much higher than most other anisotropic strain sensors (Table S1), proving them very convenient and appealing in practical full-range sensing applications. It should also be mentioned that the amplitude of ∆*R*/*R*_0_ remained the same under the same strain of 80% in both the L- and T-directions regardless of the frequency (Fig. 4b), and the electrical responses in the two directions were highly repeatable under cyclic strains ranging from 25 to 100% (Fig. 4c). The frequency-independent resistance changes verify the adaptability of the sensor to different practical scenarios.

The anisotropic electromechanical response was exploited to fabricate a multidirectional sensor by orthogonally stacking two bilayer sensors with their individual T-directions oriented same as the *X*- and *Y*-axes, respectively, (Fig. S4), enabling them to measure both the direction and magnitude of various strains arising from complex bodily movements. The individual bilayer sensor was responsible for detecting the strain components along their respective T-directions. As the strain direction to the *X*-axis increased from ${0}^{^\circ }$ to ${90}^{^\circ }$, the GF in the *X*-axis decreased and vice versa in the *Y*-axis, as shown in Fig. S24. The figure of merit of a sensor to identify the loading direction was evaluated by the selectivity, which is defined as $\Delta GF/ \theta$, where $\Delta GF$ is the GF difference between the *X*- and *Y*-axes, and $\theta$ is the loading angle from the *X*-axis (Fig. 4d). The GF, selectivity, and working range of our multidirectional sensor are compared with other anisotropic strain sensors reported in the literature (Fig. 4e and Table S1). The selectivity of our sensor was 6.3, much higher than the vast majority of existing strain sensors. The crack-based sensors are extremely sensitive with high selectivity but lack stretchability (blue region) [27, 28, 62], while the sensors based on an aligned structure exhibit ultrahigh stretchability in the alignment direction but suffer from low sensitivity (pink region) [35, 36]. For example, the sensors based on aligned CNT structures exhibited low GFs of 0.56 at small strains due to the sliding between the individual CNTs in the bundle with limited amounts of cracks created [36]. While improved sensitivity was achieved at large strains because of crack formation and propagation, resulting in exponential or bilinear responses with poor linearity. Our multidirectional sensor, however, possessed simultaneously high sensitivity, high selectivity, and a wide linear working range. Such all-inclusive performance has rarely been reported previously [37, 42, 58–65] and was made possible by rational assembly of a highly wrinkled CNT–GO film and a crack-bridged, aligned CNT film to form a bilayer composite structure.

### Application to Capture Full-range Complex Motions for Sign Language Recognition

**Fig. 5:**
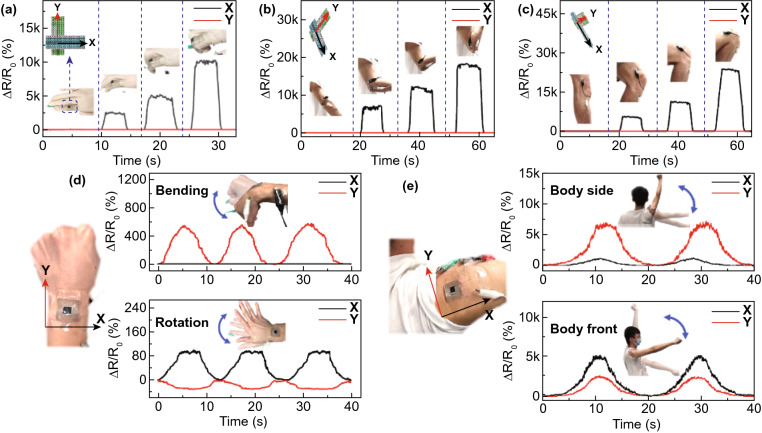
Demonstration of full-range strain sensing of complex human motions by the multidirectional sensor. Detection of bending of **a** finger, **b** elbow, and **c** knee joints to different degrees. Detection of multiaxial movements of **d** wrist and **e** shoulder joints

The multidirectional sensors (Fig. S4) were attached on the human body to demonstrate their effectiveness in measuring complex human motions ranging from subtle strains induced by pulse and voice vibration (Fig. S25) to large motions such as joint movements (Figs. 5 and S26). Based on the anisotropic electromechanical responses to strains of individual sensing units, the multidirectional sensors were able to detect not only the uniaxial bending of joints, but also multiaxial motions. When the sensors were attached to the joints of finger, elbow, and knee, the normalized resistance changes ∆*R*/*R*_0_ in the *X*-axis increased consistently with increasing bending degree while those in the *Y*-axis remained almost constant (Fig. 5a–c). Even in the deep squat exercise, the knee flexion associated with an extremely large deformation was recorded completely and accurately (Fig. 5c) because of the wide working range and linearity of the sensor. In addition to large movements, the sensor was able to distinguish subtle multiaxial joint motions, such as wrist bending and rotation, as shown in Fig. 5d. In response to the bending motion, the resistance in the *Y*-axis increased while that in the *X*-axis remained unchanged because the deformation occurred only in the *Y*-axis. For the wrist rotation, the increasing resistance in the *X*-axis and slightly negative resistance change in the *Y*-axis indicate a large tensile strain in the *X*-axis and a small compressive strain in the *Y*-axis. The multidirectional sensor was also attached on the shoulder to distinguish multiaxial motions under large movements (Fig. 5e). When raising the arm from the body side, ∆*R*/*R*_0_ in the *Y*-axis increased with increasing arm height, while that in the *X*-axis remained negligible. When the arm was raised to the body front, the signals in both axes increased with higher resistance changes, signifying the sensor’s capability to distinguish large multidirectional movements of shoulder.

**Fig. 6:**
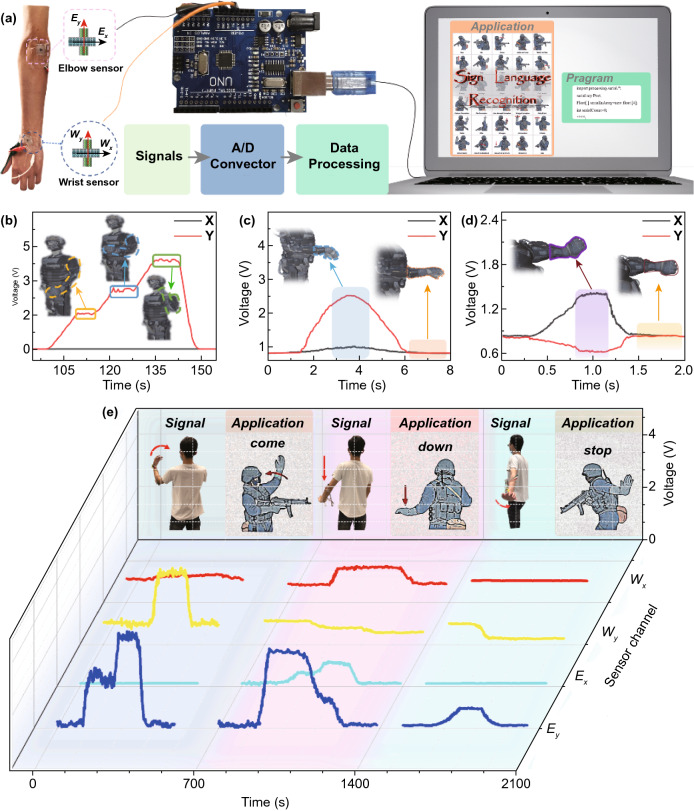
Application of the multidirectional sensor in SLRS. **a** Schematic of SLRS working principle. Manipulation of 3D character models for **b** elbow bending, **c** wrist bending, and **d** wrist rotation based on the signals generated from the multidirectional sensors attached to different parts of human body. **e** Recognition of sign languages including “come,” “down,” and “stop” based on the signals transmitted from four sensing channels

The ability to detect complicated physical motions of human joints endows our multidirectional sensor with potential applications in wearable motion captures. To demonstrate such a capability, a sign language recognition system (SLRS) was constructed using the multidirectional sensors that could distinguish different gestures in the military sign language and translate them into animations of a 3D character model. Figure 6a presents the overall working principle of the SLRS with two multidirectional sensors placed on the wrist and elbow joints each. The four sensing channels, *W*_*x*_, *W*_*y*_, *E*_*x*_, and *E*_*y*_, represent the two orthogonal sensing axes of the wrist and elbow sensors, respectively. The signals from these channels were collected and converted to digital signals, which were further transmitted to a terminal computer program. The program then analyzed the data and translated the signals to animations of an 3D character model, as shown in Fig. 6b–d. Figure 6b presents the signals from the sensor attached on the elbow joint when the arm was bent to different degrees. These signals were recognized and translated to corresponding actions of the 3D model. Similarly, the bending and rotation of wrists were also correctly converted to corresponding aminations, as shown in Fig. 6c, d. In addition to the movements of wrists and elbows, large deformation generated by deep squat was successfully recognized by attaching the sensors on the knee and translated to the same action of the 3D model (Fig. S26 and Video S3).

The accurate responses of individual sensing units through four channels allowed the SLRS to translate diverse actions to animations accompanied by text messages. Typical examples of distinguishing the sign languages of “come,” “down,” and “stop” and translating them to animated and text messages are shown in Fig. 6e. The SLRS could accurately identify the sign languages by differing signals from four sensing channels both in their magnitudes and patterns. For example, in the “come” action, the signals from the *W*_*x*_ and *E*_*x*_ channels remained low while the *W*_*y*_ and *E*_*y*_ channels experienced a drastic increase in voltage. By contrast, in the “down” action, the *W*_*x*_ and *E*_*x*_ channels showed slightly increased signals, while the signals from the W_y_ channel decreased and *E*_*y*_ channel increased greatly to around 3 V. The whole course of recognition and translation of the above sign languages is included in Video S4. It should be noted that the sharp difference in the signals implies the high sensitivity and selectivity of each anisotropic sensor. Coupled with a wide linear working range, the multidirectional sensor holds tremendous potentials in computer animation, interactive video games, and human–machine interfacing.

## Conclusion

In summary, we developed anisotropic strain sensors consisting of a periodically wrinkled and microcracked CNT–GO hybrid film at the bottom and a top-aligned CNT film. The rationally assembled bilayer sensor exploited the merits of the two anisotropic structures to achieve highly selective and ultrasensitive capability with an ultrawide working range and excellent linearity, as summarized below.

1. Periodic wrinkles were created aiming at maintaining extremely low sensitivity in the L-direction, while the microcracks in the bottom layer were bridged by the top-aligned CNTs ensuring high sensitivity to strains in the T-direction. Such anisotropic sensing behavior with an ultralarge GF difference between the orthogonal directions, namely GFs of 0.15 and 287.6 in the L- and T-directions, respectively, brought about ultrahigh selectivity of 6.3.
2. High stretchability in the T-direction was achieved thanks to the stepwise crack propagation mechanism in the crack-bridging bilayer structure, contributing to a wide working range of 100% strain.
3. The synergistic cross-influence between the top-aligned CNTs and the wrinkled and cracked bottom CNT–GO film gave rise to excellent linearity over the entire working range of 100 % strain. The synergy was enhanced by tuning the area ratio of the top to bottom films, which in turn moderated the crack propagation at high strains.

Multidirectional sensors were assembled by orthogonally stacking two bilayer films, which demonstrated accurate detection of both the amplitudes and directions of strains generated by complex full‐range human motions. These exceptional sensing capabilities of the multidirectional sensor would find potential applications in wearable motion capture devices for emerging wearable electronics and artificial skins.

## Supplementary Information

Below is the link to the electronic supplementary material.

Supplementary file1 (MP4 11920 KB)

Supplementary file2 (MP4 16667 KB)

Supplementary file3 (MP4 4069 KB)

Supplementary file4 (PDF 1881 KB)

Supplementary file5 (ZIP 1848 KB)
