# Supplementary material for: Anisotropic, Wrinkled, and Crack-Bridging Structure for Ultrasensitive, Highly Selective Multidirectional Strain Sensors
Source: Nanomicro Lett. 2021 May 4;13:122. doi: 10.1007/s40820-021-00615-5 (PMC8096879; doi:10.1007/s40820-021-00615-5)
Supplement: Supplementary file 4 — Supplementary file4 (PDF 1881 KB) [file 40820_2021_615_MOESM4_ESM.pdf]

Supporting Information for

Anisotropic Wrinkled and Crack-Bridging Structure for Ultrasensitive, Highly Selective Multidirectional Strain Sensors

Heng Zhang[1], Dan Liu[1], Jeng-Hun Lee[1], Haomin Chen[1], Eunyoung Kim[1], Xi Shen[1,]*, Qingbin Zheng[1, 2,]*, Jinglei Yang[1,]*, Jang-Kyo Kim[1,]*

[1]Department of Mechanical and Aerospace Engineering, The Hong Kong University of Science and Technology, Clear Water Bay, Kowloon, Hong Kong, P. R. China

[2]School of Science and Engineering, The Chinese University of Hong Kong, Shenzhen, Guangdong 518172, P. R. China

*Corresponding authors. E-mail: maeshen@ust.hk (Xi Shen); zhengqingbin@cuhk.edu.cn (Qingbin Zheng); maeyang@ust.hk (Jinglei Yang); mejkkim@ust.hk (Jang-Kyo Kim)

S1 Materials and Methods

S1.1 Fabrication of CNT-GO Hybrid Film

The CNT solution was prepared by dispersing 5 mg CNTs in 100 mL deionized water with the aid of 1 g Triton X-100 surfactant under sonication (Sonic VCX 750) at 400 W for 30 min [S1]. The suspension was kept in an ice bath to avoid heating during sonication. The GO dispersion of 0.5 mg mL^{-1} was prepared following the modified Hummers method [S2]. Five grams of natural graphite flakes were treated with a mixture of sulfuric acid (150 mL) and nitric acid (50 mL), followed by thermal expansion at 1050 °C for 15 s. The expanded graphite was oxidized by $KMnO_4$ (10 mg) and H_2O_2 (50 mL) in 200 mL sulfuric acid. The collected GO solution was centrifuged and diluted with deionized water. The CNT-GO hybrid film was obtained by vacuum infiltration of the CNT and GO mixture (at a mass ratio of 1:1) on a cellulose membrane (pore size 0.45 μm, N8645-100EA, SIGMA).

S1.2 Fabrication of Vertically Aligned CNT Forest

Vertically aligned CNT forests of 500 μm in height were synthesized by water-assisted chemical vapor deposition (CVD) method [S3]. The Al_2O_3 (40 nm)/Fe (1 nm) catalyst was sputtered on silicon wafers. The precursor gas C_2H_4 (75 sccm) and water (100 ppm) were fed into a CVD tube using He and H_2 as carrier gases at a feeding rate of 1 L min^{-1}. The tube was heated to 750 °C at a rate of 100 °C min^{-1} and kept for 30 min before cooling down to room temperature to allow the growth of CNT forests.

S1.3 Calculation of Interaction Energies

To calculate the interaction energy between the individual CNTs, a simplified molecular structure containing three identical CNTs with a diameter of 7.2 Å and length of 32 Å were used, as shown in Fig. 3c. To calculate the interaction energy between the 1D CNT and 2D GO sheet, one CNT in the above model was replaced by a GO sheet of the same atomic weight. The interaction energy was calculated by:

$$E_{interaction} = |E_{total} - (E_{CNT} + E_{GO})| \qquad \text{(S1)}$$

where E_{total}, E_{CNT} and E_{GO} are the energies of the whole system, CNTs without GO, GO without CNTs, respectively.

To calculate the interaction energy between the bottom CNT-GO hybrid film and the top aligned CNT array, a bilayer structure consisting of (i) a layer of seven aligned CNTs and (ii) a layer of CNT-GO random mixture with a lateral dimension of 75 × 80 Å was constructed (insets in Fig. 3d). The periodic boundary conditions were applied in the plane directions while a vacuum slab of 200 Å was built in the thickness direction to avoid interactions between the periodic images. A thin layer of CNT and GO random mixtures at a weight ratio of 1:1 was built using the Amorphous Cell module followed by energy minimization to establish an equilibrium state. The interaction energy between the two layers was calculated similarly according to Eq. S1.

As for the aligned CNTs with PDA treatment, a layer of PDA molecules was first constructed using the Amorphous Cell module with a target density of 1.54 g cm^{-3} according to the Swift's theoretical model [S4]. The molecular structures of dopamine monomers shown in Fig. S16 were used to construct PDA [S5]. The obtained PDA layer was attached to the bottom surface of aligned CNTs, followed by stacking with a CNT-GO hybrid layer, as shown in Fig. 3d. The interaction energy between the CNT-PDA and CNT-GO layers was calculated according to Eq. S1.

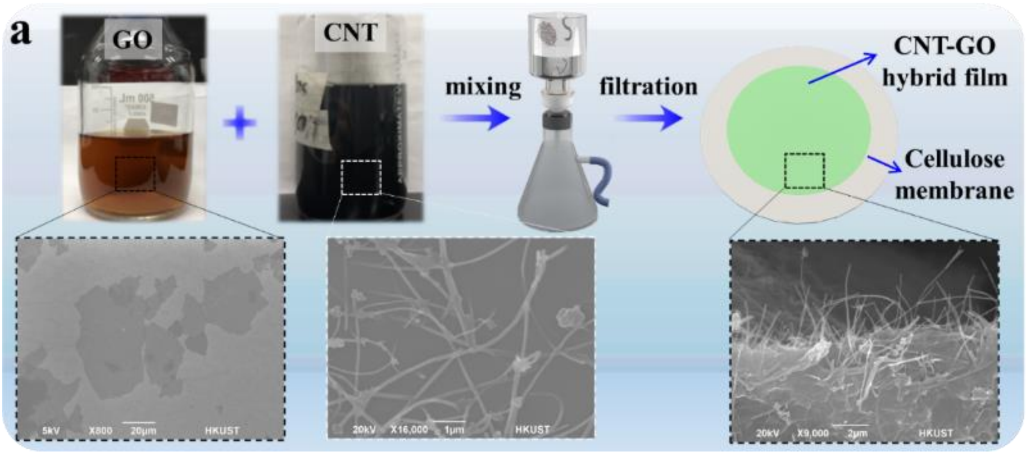

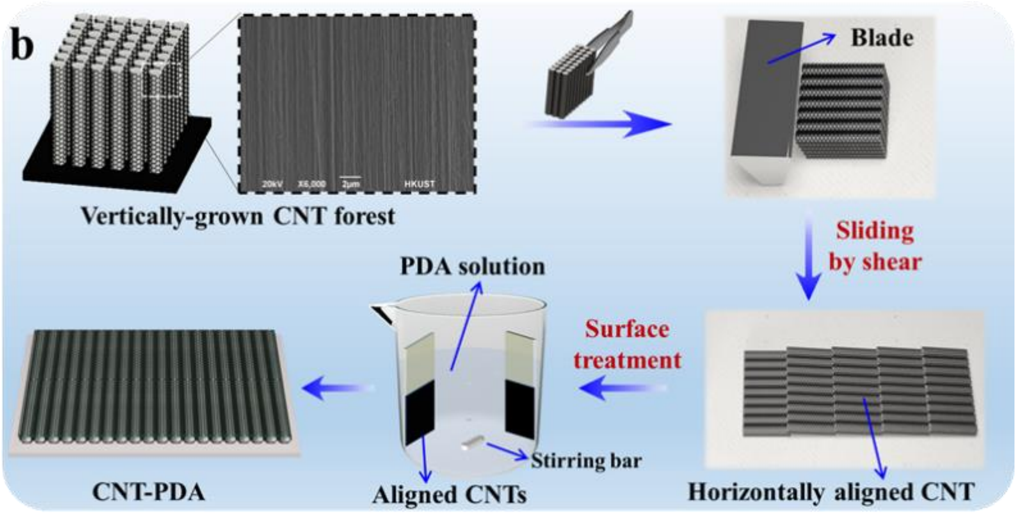

Fig. S1 Fabrication of (**a**) CNT-GO hybrid film and (**b**) CNT-PDA film. SEM images of GO sheets, randomly-distributed CNTs, CNT-GO hybrid film and vertically-grown CNT forest in inset

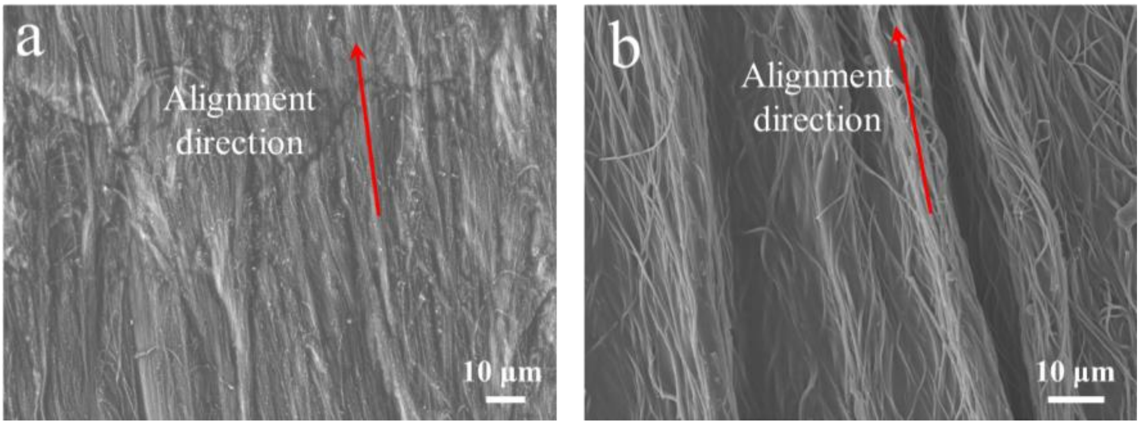

Fig. S2 Morphologies of aligned CNT structures (**a**) before and (**b**) after PDA treatment and transfer to the CNT-GO hybrid film

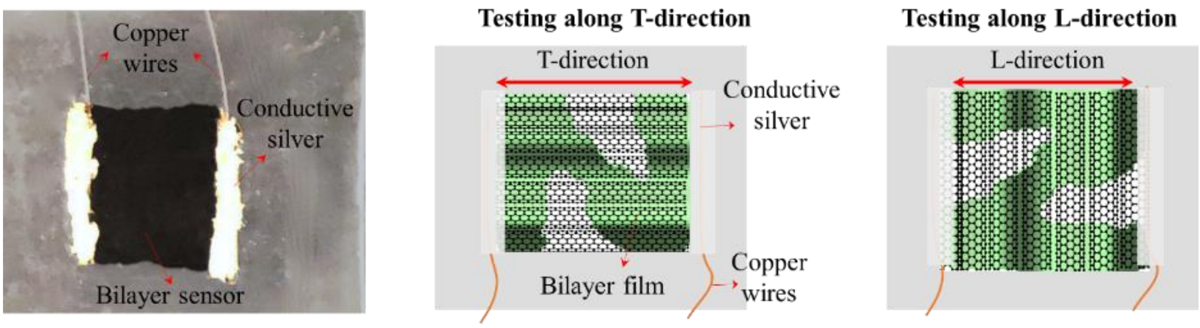

Fig. S3 Photographs and schematics showing the connection of copper wires in the bilayer sensor when tested along L- and T-directions

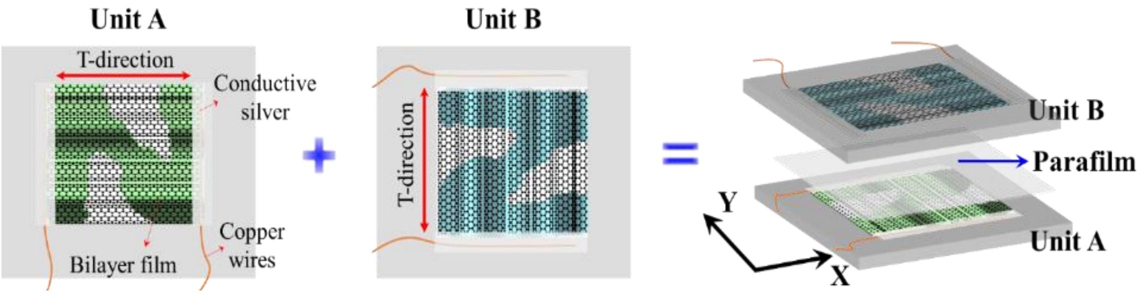

Fig. S4 Assembly of a multidirectional sensor using two orthogonally-stacked bilayer sensors, units A and B. The copper wires were connected to the two edges along their respective T-directions to simultaneously detect the strain components along their respective T-directions (i.e., X- and Y-axis)

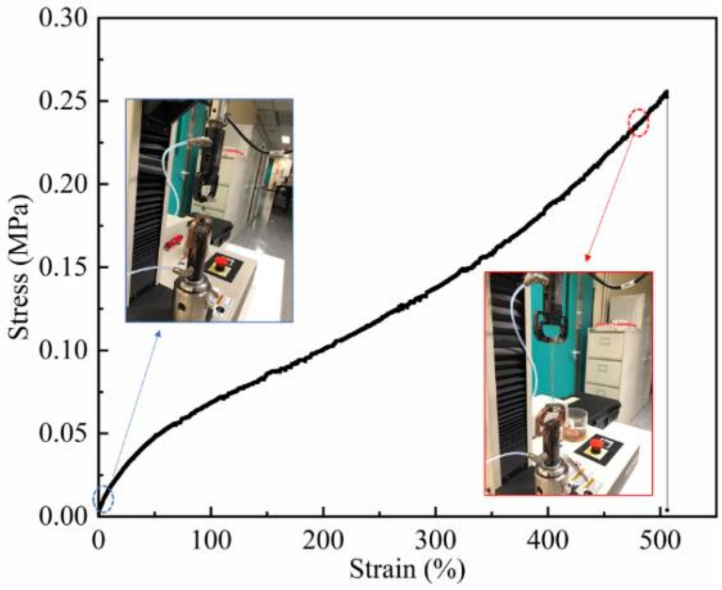

Fig. S5 Strain-stress curve of the strain sensor under uniaxial tension showing a failure strain of over 500% at room temperature

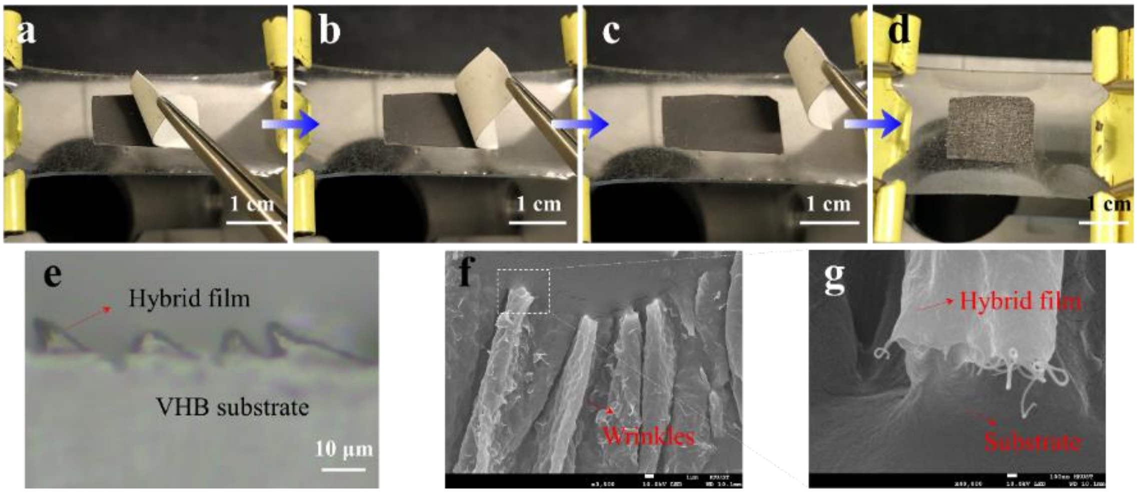

Fig. S6 Strong adhesion between CNT-GO hybrid film and elastomer substrate. (**a-c**) The membrane was easily peeled off after transferring the hybrid film to the substrate,

indicating the adhesion between the substrate and hybrid film is much stronger than that between the membrane and hybrid film. (**d**) Release of pre-strain to create wrinkles. (**e**) Cross-sectional optical image of the wrinkled film on the substrate showing good connection between the two without gaps. Top-view SEM images of the wrinkled film on the substrate at (**f**) low and (**g**) high magnifications

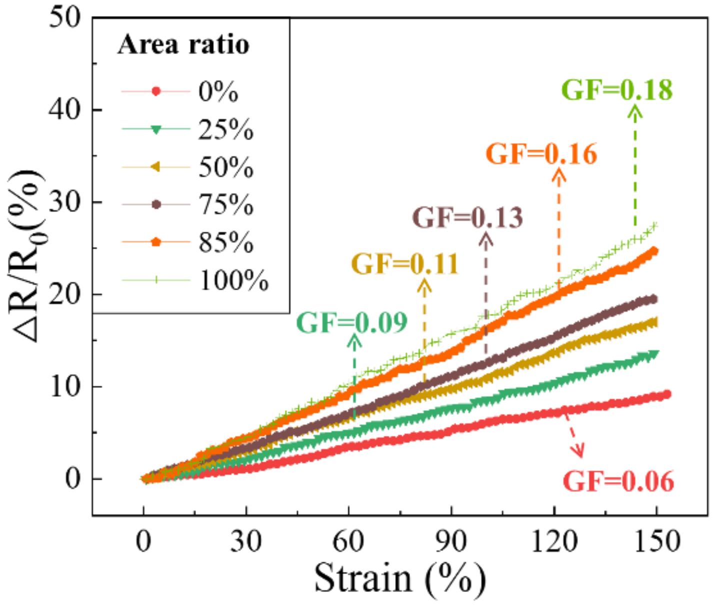

Fig. S7 Relative resistance changes of the sensors with different area ratios at strains applied in the L-direction

S2 Equivalent Circuit Model for Bilayer Sensor Loaded in T-direction

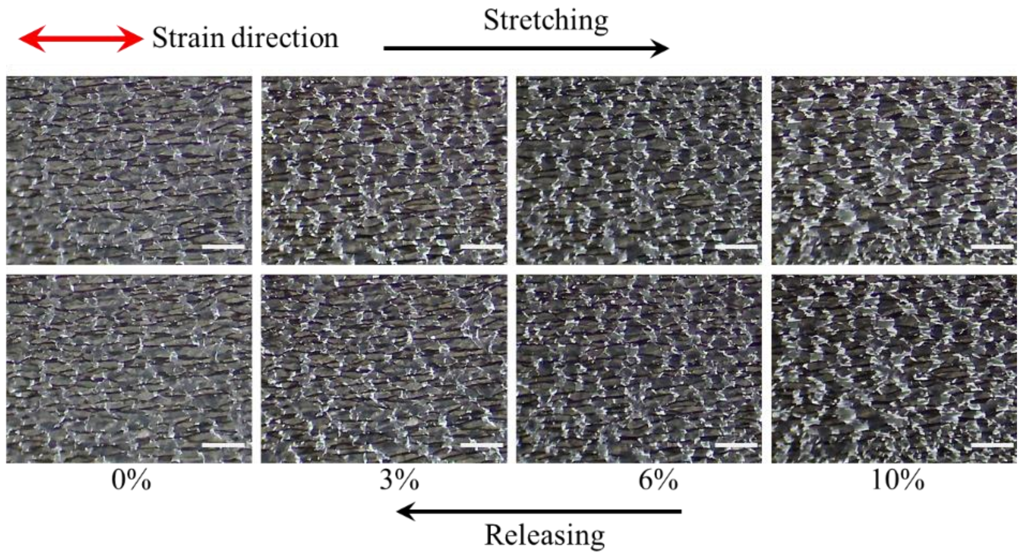

Fig. S8 Morphological changes of the bottom CNT-GO film when loaded in T-direction. Scale bars: 30 μm

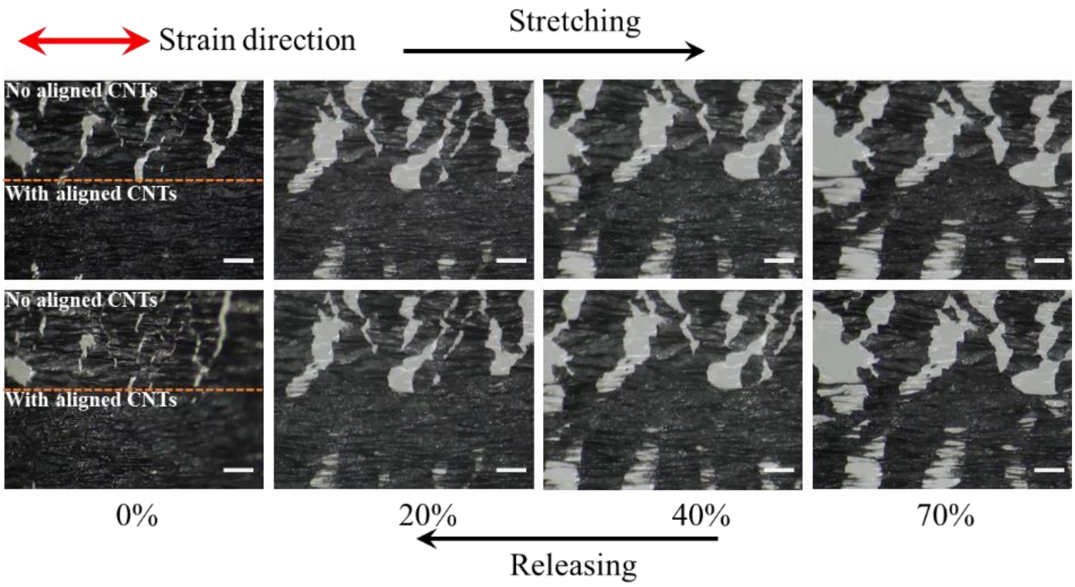

Fig. S9 Strain-dependent morphological changes of the bilayer sensor when loaded in T-direction. Scale bars: 20 μm

The electromechanical performance of the bilayer sensor in the T-direction is influenced by the stepwise crack propagation mechanism. At a relatively small strain, the cracks initiated and propagated in the bottom CNT-GO film, leading to a sharp increase in resistance. Meanwhile, the top aligned CNT arrays remained intact with only slight sliding between the CNT bundles. A schematic of a cracked region in the bilayer film and the corresponding equivalent circuit model are shown in Fig. S10. The electrons transported between the two fragments *via* two parallel paths, namely, (i) tunneling through the cracks in the bottom layer; and (ii) CNT bridges connecting the two fragments in the top layer. The overall resistance of the bilayer film consists of numerous such cracked regions in series. Thus, the normalized resistance change is given by:

$$\frac{\Delta R}{R_0} = \frac{R_i - R_0}{R_0} = \sum_{i=1}^{n_b} \frac{1}{R_{0,i}} / \left(\frac{1}{R_{bcrack,i}} + \frac{1}{R_{bc,i} + R_{CNT,i}}\right) - 1 = \frac{\sum_{i=1}^{n_b} \frac{R_{bcrack,i} \times (R_{bc,i} + R_{CNT,i})}{R_{bcrack,i} + R_{bc,i} + R_{CNT,i}}}{\sum_{i=1}^{n_b} R_{0,i}} - 1$$

(S2)

where $R_{0,i}$ is the initial resistance of the bilayer film, $R_{bcrack,i}$ is the tunneling resistance, $R_{bc,i}$ is the contact resistance between the top and bottom layers, $R_{CNT,i}$ is the resistance of the aligned CNT bundle. It is noted that these parameters represent the resistances of a unit crack shown in Fig. S10, and n_b is the number of such units interconnected in series to yield the overall resistance.

The tunneling resistance is dependent on the applied strain, ε, and given by [6]:

$$R_{bcrack,i} = a d_0 \varepsilon \times e^{b d_0 \varepsilon} \qquad \text{(S3)}$$

where d_0 is the initial tunneling distance without strain, $a = \frac{h^2}{A_j e^2 \sqrt{2qE_c}}$ and $b = \frac{4\pi}{h}\sqrt{2qE_c}$. Here, h is the Plank's constant, A_j is the junction area, q is a constant structural parameter related to the fractal structure of the network, and E_c is the height of the potential barrier.

The contact resistance is assumed constant independent of applied strain, namely, $R_{bc,i} = \alpha_{ic}$ because no obvious change occurs in contact area between the top and bottom layers during stretching due to the strong interlayer adhesion [S7, S8].

The resistance of the CNT bundle is a function of applied strain:[8]

$$R_{CNT,i} = \frac{\rho_C(l_0\varepsilon + l_0)}{A_C} \tag{S4}$$

where ρ_C is the intrinsic resistivity of the aligned CNT bundle, l_0 is the initial length of the CNT bundle without strain, and A_C is the cross-sectional area of the CNT bundle.

To simplify the model, we assume the resistances in the individual units are the same, namely:

$R_{bcrack,1} = R_{bcrack,2} = \cdots = R_{bcrack,n}$;

$R_{bc,1} = R_{bc,2} = \cdots = R_{bc,n}$;

$R_{CNT,1} = R_{CNT,2} = \cdots = R_{CNT,n}$.

Based on the above, Eq. S2 is rewritten as:

$$\frac{\Delta R}{R_0} = \frac{\dfrac{R_{bcrack,i} \times (R_{bc,i} + R_{CNT,i})}{R_{bcrack,i} + R_{bc,i} + R_{CNT,i}}}{R_{0,i}} - 1$$

$$= \frac{\left(\frac{\rho_c l_0 \varepsilon}{A_C} + \frac{\rho_c l_0}{A_C} + \alpha_{ic}\right) \times a d_0 \varepsilon \times e^{b d_0 \varepsilon}}{R_{0,i}\left(\frac{\rho_c l_0 \varepsilon}{A_C} + \frac{\rho_c l_0}{A_C} + \alpha_{ic} + a d_0 \varepsilon \times e^{b d_0 \varepsilon}\right)} - 1$$

$$= \frac{(A + B\varepsilon) \times C\varepsilon e^{D\varepsilon}}{A + B\varepsilon + C\varepsilon e^{D\varepsilon}} - 1 = f(\varepsilon) \tag{S5}$$

In Eq. S5, the normalized resistance change at small strains is expressed as a function of strain, $f(\varepsilon)$, which is used to fit the experimental data using four strain-independent non-dimensional coefficients, A=$(\alpha_{ic} + \frac{\rho_c l_0}{A_C})/R_{0,i}^{'}$, $B = \frac{\rho_c l_0}{A_c}/R_{0,i}^{'}$, $C = a d_0/R_{0,i}^{'}$, and $D = b d_0$.

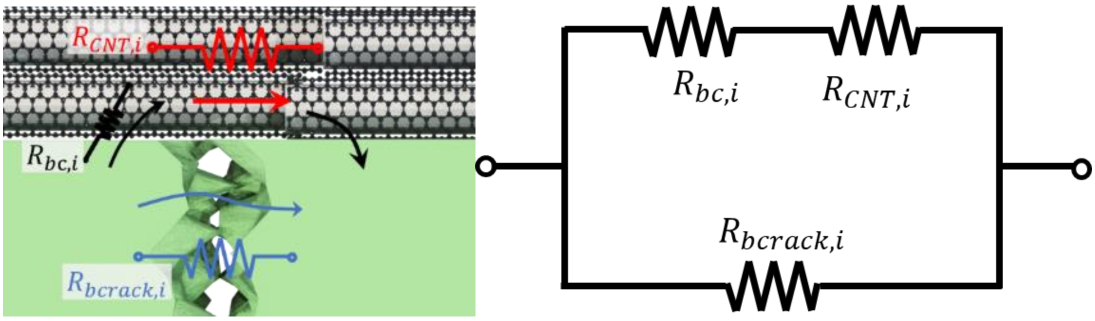

Fig. S10 Schematic illustration of sensing mechanism in T-direction at small strains and the corresponding equivalent circuit model

With increasing strain, the cracks in the bottom layer became too large to allow tunneling. Meanwhile, the sliding between the CNT bundles generated cracks in the top aligned CNT layer, which became gradually more important in increasing the resistance. The electron transports in the top layer and the corresponding equivalent circuit model are shown in Fig. S11. Some electrons are transferred through tunneling between the disconnected CNTs, while others are transferred to the adjacent CNTs by physical contacts.

Therefore, the resistance variation caused by the crack propagation in the aligned CNT layer is given by:

$$\frac{\Delta R}{R_0} = \sum \frac{1}{R_{0,i}} / \left(\frac{1}{R_{tcrack,i}} + \frac{1}{R_{cc,i}+R_{CNT,i}}\right) - 1 = \frac{\sum_{i=0}^{m'} \frac{R_{tcrack,i}\times(R_{cc,i}+R_{CNT,i})}{R_{tcrack,i}+R_{cc,i}+R_{CNT,i}}}{\sum_{i=0}^{n'} R_{0,i}} - 1 \qquad \text{(S6)}$$

where n' is the total number of initial cracks, m' is the number of cracks under applied strains, $R_{tcrack,i}$ is the tunneling resistance through the cracks, $R_{cc,i}$ is the contact resistance between the adjacent CNTs.

Assuming the same type of resistance in each unit has the same magnitude, Eq. S11 can be simplified as:

$$\frac{\Delta R}{R_0} = \frac{m'}{n'} \times \frac{\frac{R_{tcrack,i}\times(R_{cc,i}+R_{CNT,i})}{R_{tcrack,i}+R_{cc,i}+R_{CNT,i}}}{R_{0,i}} - 1 \qquad \text{(S7)}$$

It is assumed that m' is proportional to the applied strain, ε, because a larger strain leads to more cracks in the film. Therefore, we can write $m'/n' = k'\varepsilon$, where k' is the failure probability [6, 9].

Using the similar treatment as in Eqs. S2-S5 yields:

$$\frac{\Delta R}{R_0} = k'\varepsilon \times \frac{(A'+B'\varepsilon)\times C'\varepsilon e^{D'\varepsilon}}{A'+B'\varepsilon+C'\varepsilon e^{D'\varepsilon}} - 1 = g(\varepsilon) \qquad \text{(S8)}$$

Combining Eqs. S5 and S8, the overall normalized resistance change can be written depending on the applied strain as:

$$\frac{\Delta R}{R_0} = \begin{cases} f(\varepsilon) & 0 \leq \varepsilon < \varepsilon_t \\ g(\varepsilon) & \varepsilon_t \leq \varepsilon < \varepsilon_w \end{cases} \quad \text{(S9)}$$

where ε_t is the transition strain which separate the two regions in the relative resistance change curves and ε_w is the working range which is around 100% strain for the optimized-structure.

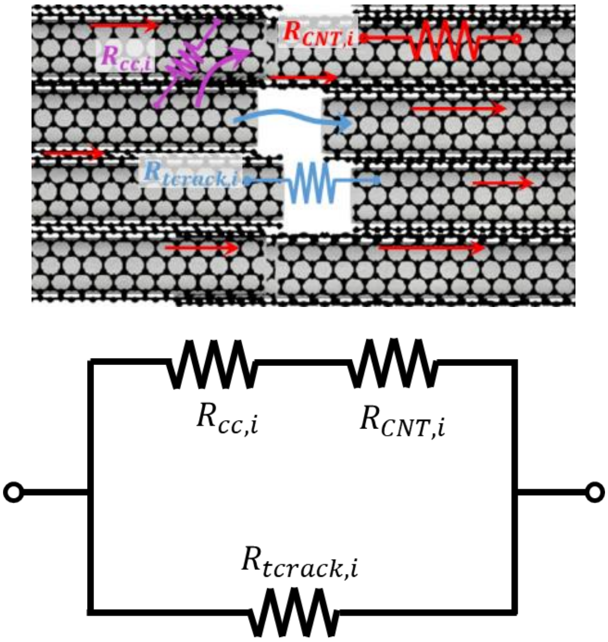

Fig. S11 Schematic illustration of sensing mechanism of crack propagation in the top CNT film and the corresponding equivalent circuit model

The coefficients *A* to D' are determined by fitting the experimental data for the two sensors with 50% and 85% area ratios and a constant 25% GO content according to Eq. S9, as shown in Fig. S12 and Table S2. The bilinear responses are in reasonable agreement with $f(\varepsilon)$ at small strains and $g(\varepsilon)$ at large strains. The nonlinear behavior for the former sensor with 50% area ratio and 25% GO content (Fig. S12a) is due to different crack propagation rates in two regions. An effective method to achieve good linearity across the whole working range is the reduction of the slope of $g(\varepsilon)$ by tuning the area ratio, so that the crack propagation at small and large strains are matched. Specifically, increasing the area ratio from 50% to 85% yields lower B', C', D' and k' values, suggesting moderate crack propagation through CNT bridging at large strains. Therefore, an almost linear response was achieved for the sensor with an area ratio of 85% (Fig. S12b). The above analysis highlights the possibility of tailoring the linearity of sensors by matching the crack propagation at both small and large strains via tuning the material parameters.

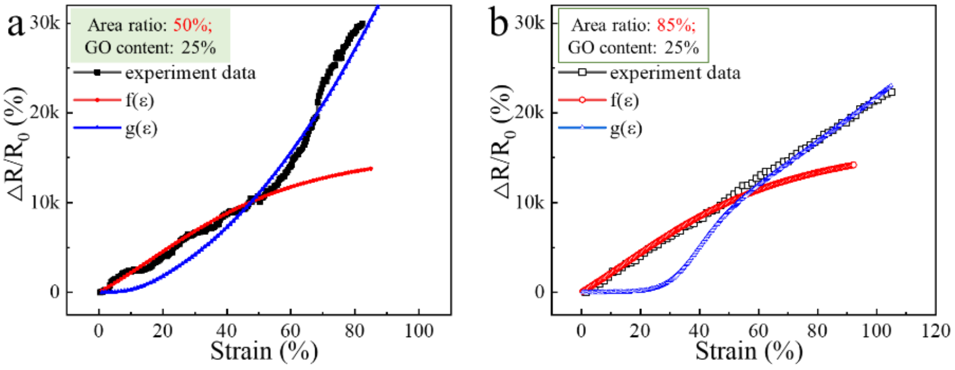

Fig. S12 Relative resistance changes in T-direction of the sensors prepared with (a) 50% area ratio and 25% GO content and (b) 85% area ratio and 25% GO content, which are fitted with $f(\varepsilon)$ and $g(\varepsilon)$

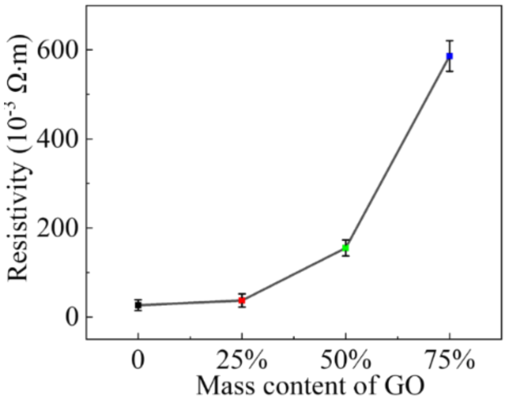

Fig. S13 Resistivities of CNT-GO hybrid films with different GO mass contents

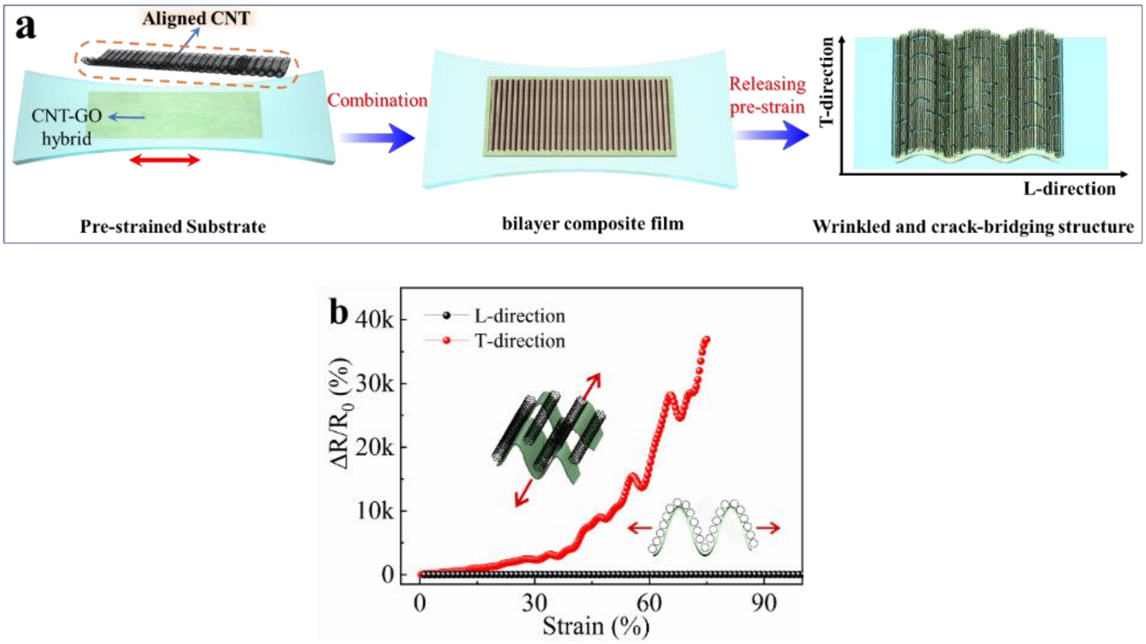

Fig. S14 (**a**) Schematic of the fabrication of a sensor (with an area ratio of 85% and a GO content of 50%) without a pre-cracking step by directly covering the aligned CNTs on stretched CNT-GO film. (**b**) Relative resistance changes in L- and T-directions of the sensor

The sensor exhibited a nonlinear response with stretchability of 75%, which is much inferior to the sensor fabricated with the pre-cracking step having a highly linear response with high stretchability of 100% (Fig. 4a).

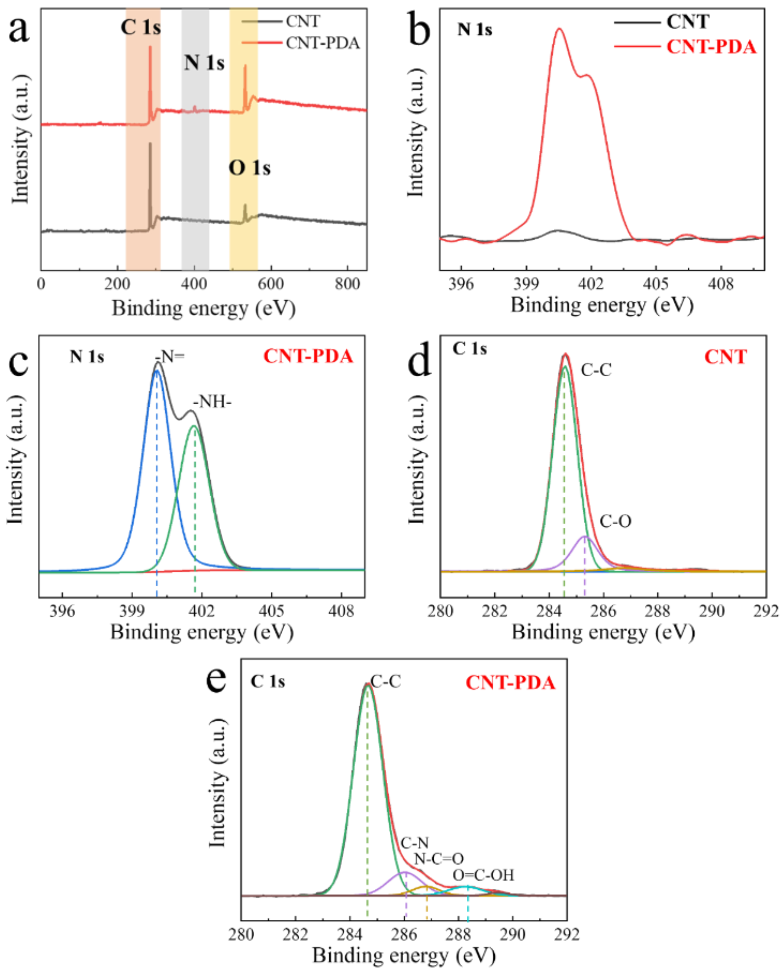

Fig. S15 (**a**) XPS spectra of CNTs before and after PDA treatments. The CNT-PDA had a discernable N1s signal, indicating PDA coating on CNTs. (**b**) XPS N1s spectra of CNTs and CNT-PDA. (**c**) Deconvoluted XPS N1s spectra of CNT-PDA. Deconvoluted XPS C1s spectra of (d) CNTs and (**e**) CNT-PDA

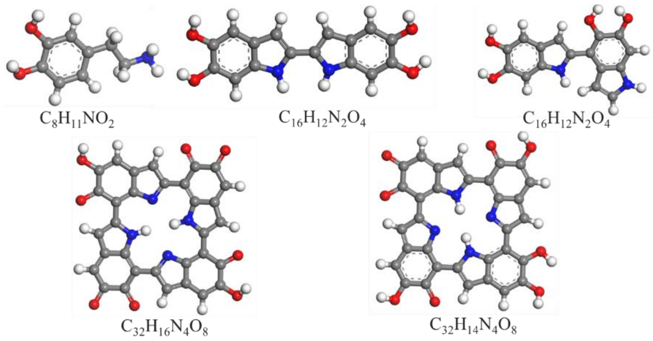

Fig. S16 Representative molecular structures of dopamine monomers used to construct PDA for treatment of aligned CNTs [S5]. C atoms in grey, H in white, N in blue, and O in red

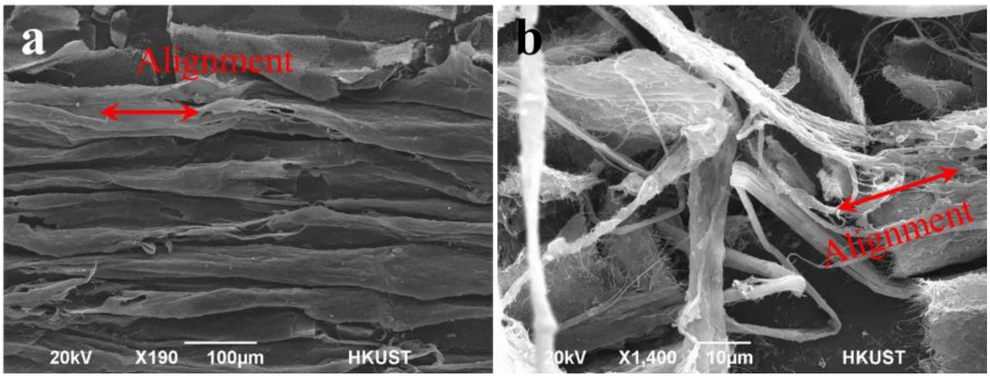

Fig. S17 Morphologies of bilayer films (**a**) with and (**b**) without PDA treatments after 100 stretching cycles. The red arrows show the alignment direction of top CNT layer.

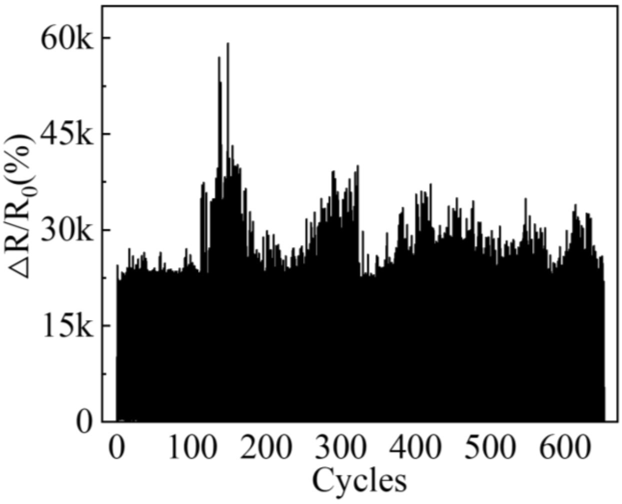

Fig. S18 Fluctuation in resistance changes of sensors without PDA treatment under cyclic 80% strain

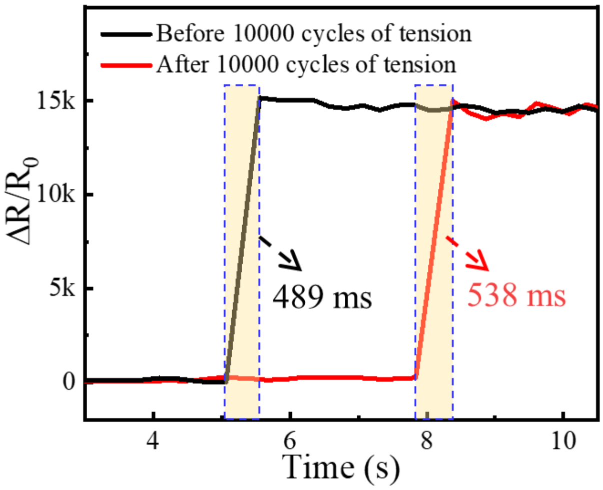

Fig. S19 Response time of bilayer sensor before and after 10000 cycles of tension

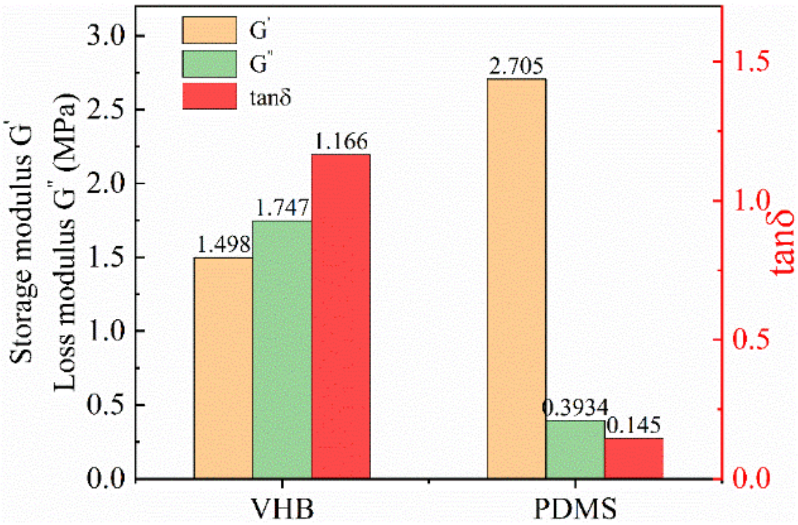

Fig. S20 Comparison of storage modulus and loss moduli between VHB and PDMS

The PDMS (Sylgard184, Dow corning) samples were prepared by mixing the base and curing agents at a ratio of 10:1. The storage and loss moduli were measured using a Rheometer (MCR 301) at room temperature (25 °C) and a constant frequency of 1 Hz. The loss modulus of VHB was higher than its storage modulus, leading to a high tan of 1.166. In contrast, the tan of PDMS was only 0.145, indicating a less viscous behavior than VHB. The viscous nature of VHB substrates resulted in a sluggish response to the applied load, contributing to a slightly longer response time of ~500 ms of our sensor than ~ 130 ms for those using a PDMS substrate.

S3 Equivalent Circuit Model for Bilayer Sensor Loaded in L-direction

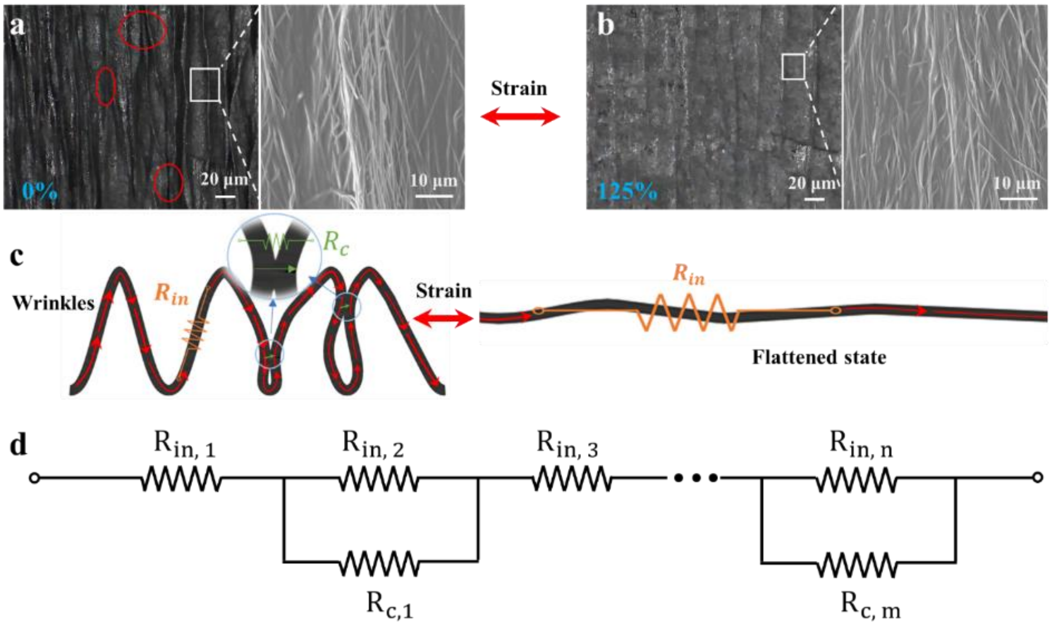

Fig. S21 Sensing mechanism in L-direction. Morphologies of bilayer sensors (**a**) before and (**b**) after 125% strain in L-direction. The red circles in (**a**) indicate the contacting crests. (**c**) Schematic illustration of straightening of wrinkles and corresponding resistance change in tension. (**d**) Equivalent circuit model

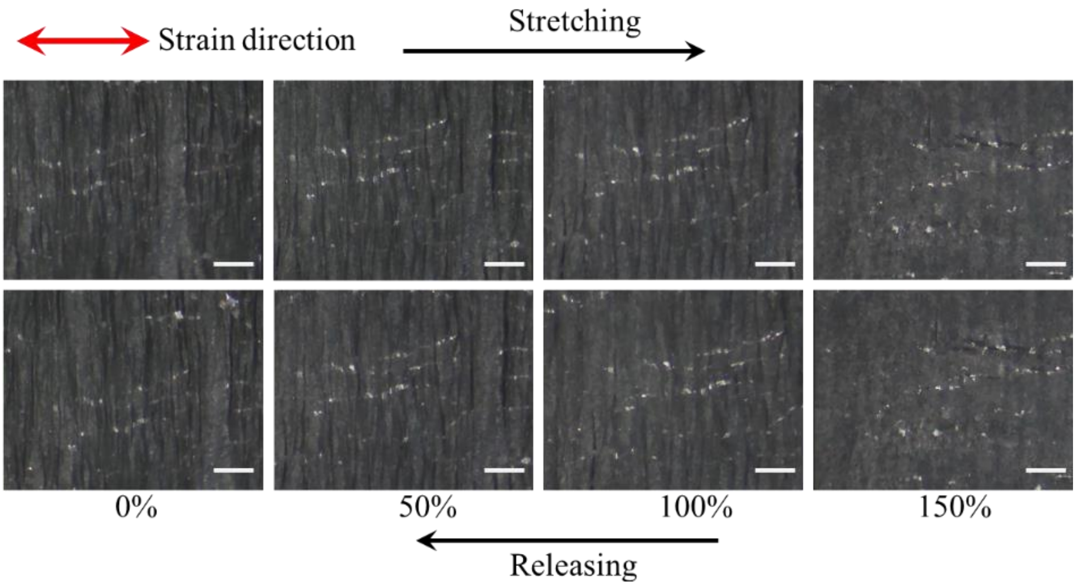

Fig. S22 Morphological changes of the bilayer sensor when loaded in L-direction. Scale bars: 20 μm

The tensile strain applied in the L-direction expended only for flattening the wrinkles without altering the conductive networks of the entire sensor, causing negligible resistance changes. Nonetheless, the adjacent crests arising from buckling may contact with each other, affording electron tunneling and thus providing additional conducting paths. Two different types of resistors exist in the equivalent circuit, namely, the resistance of bilayer film, R_{in}, and the contact resistance between the contacting crests, R_c. The two resistors are arranged in parallel [S10]. Then, the initial resistance, R_0, is given by:

$$R_0 = \sum_{i=1}^{n-m} R_{in,i} + \sum_{j=1}^{m} \frac{R_{in,j} \times R_{c,j}}{R_{in,j} + R_{c,j}} \qquad \text{(S10)}$$

where $R_{in,i}$ is the resistance of bilayer film, $R_{c,j}$ is the contact resistance between the wrinkle crests, n and m are the total number of $R_{in,i}$ and $R_{c,j}$ within the conductive network, respectively.

When the tensile strain is applied, the wrinkles gradually flatten and the number of contacts between the crests decreased. Assuming the number of contacts between crests decreased to m_i at a strain ε, the normalized resistance change, $\Delta R/R_0$, is given by:

$$\frac{\Delta R}{R_0} = \frac{\sum_{i=1}^{m-m_i}\left(R_{in,i} - \frac{R_{in,i} \times R_{c,i}}{R_{in,i} + R_{c,i}}\right)}{\sum_{i=1}^{n-m} R_{in,i} + \sum_{j=1}^{m} \frac{R_{in,j} \times R_{c,j}}{R_{in,j} + R_{c,j}}} \qquad \text{(S11)}$$

To simplify the quantitative analysis, we assume the resistance of the same type is equal, namely, $R_{in,1} = R_{in,2} = \cdots = R_{in,n}$ and $R_{c,1} = R_{c,2} = \cdots = R_{c,m}$. Then, Eq. S11 can be further simplified to:

$$\frac{\Delta R}{R_0} = \frac{(m-m_i)\times R_{in,i}{}^2/(R_{in,i}+R_{c,i})}{(n-m)R_{in,i}+m\times\frac{R_{in,j}\times R_{c,j}}{R_{in,j}+R_{c,j}}} = \frac{(m-m_i)\times R_{in,i}}{(n-m_0)(R_{in,i}+R_{c,i})+m_0\times R_{c,j}} \quad \text{(S12)}$$

Since the value of contact resistance is much larger than that of the intrinsic resistance, i.e., $R_{c,j} \gg R_{in,i}$, their sum can be approximated, $R_{in,i} + R_{c,i} \approx R_{c,i}$. Eq. S12 is therefore simplified to:

$$\frac{\Delta R}{R_0} = \frac{(m-m_i)\times R_{in,i}}{(n-m_0)R_{c,i}+m_0\times R_{c,j}} = \frac{(m-m_i)\times R_{in,i}}{n\times R_{c,j}} \quad \text{(S13)}$$

It is worth noting that $(m - m_i)/n$ is the reduction rate of the number of contacts, which is a probability function of the strain ε:

$$\frac{(m-m_i)}{n} = \phi\varepsilon \quad \text{(S14)}$$

where ϕ is a scale parameter and can be described by the Weibull empirical distribution function [11]. In this work, we assume it has a fixed value of 0.5 to perform the linear fitting [12].

Combining Eqs. S13 and S14 gives:

$$\frac{\Delta R}{R_0} = \phi\frac{R_{in,i}}{R_{c,j}} \times \varepsilon \quad \text{(S15)}$$

For the fitting purpose, Eq. S15 is expressed as:

$$\frac{\Delta R}{R_0} = \phi k_p \times \varepsilon \quad \text{(S16)}$$

where $k_p = R_{in,i}/R_{c,j}$. The experiment data were fitted with Eq. S16, as shown in Fig. S23. The value k_p obtained from the slope of the fitted line was 0.296, suggesting the much lower intrinsic resistance ($R_{in,i}$) than the contact resistance ($R_{c,j}$), which was responsible for the low GF in the L-direction.

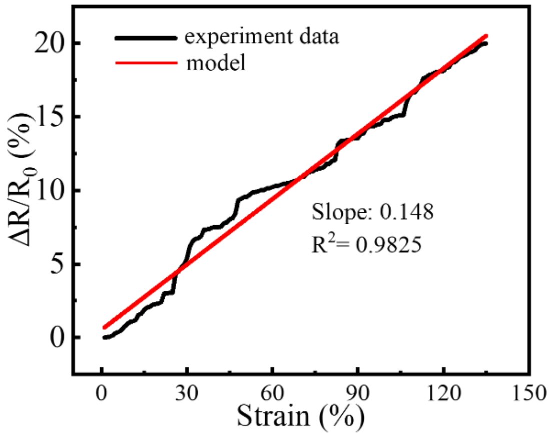

Fig. S23 Experimental data and corresponding linear fitting of relative resistance changes of the anisotropic sensor loaded in L-direction

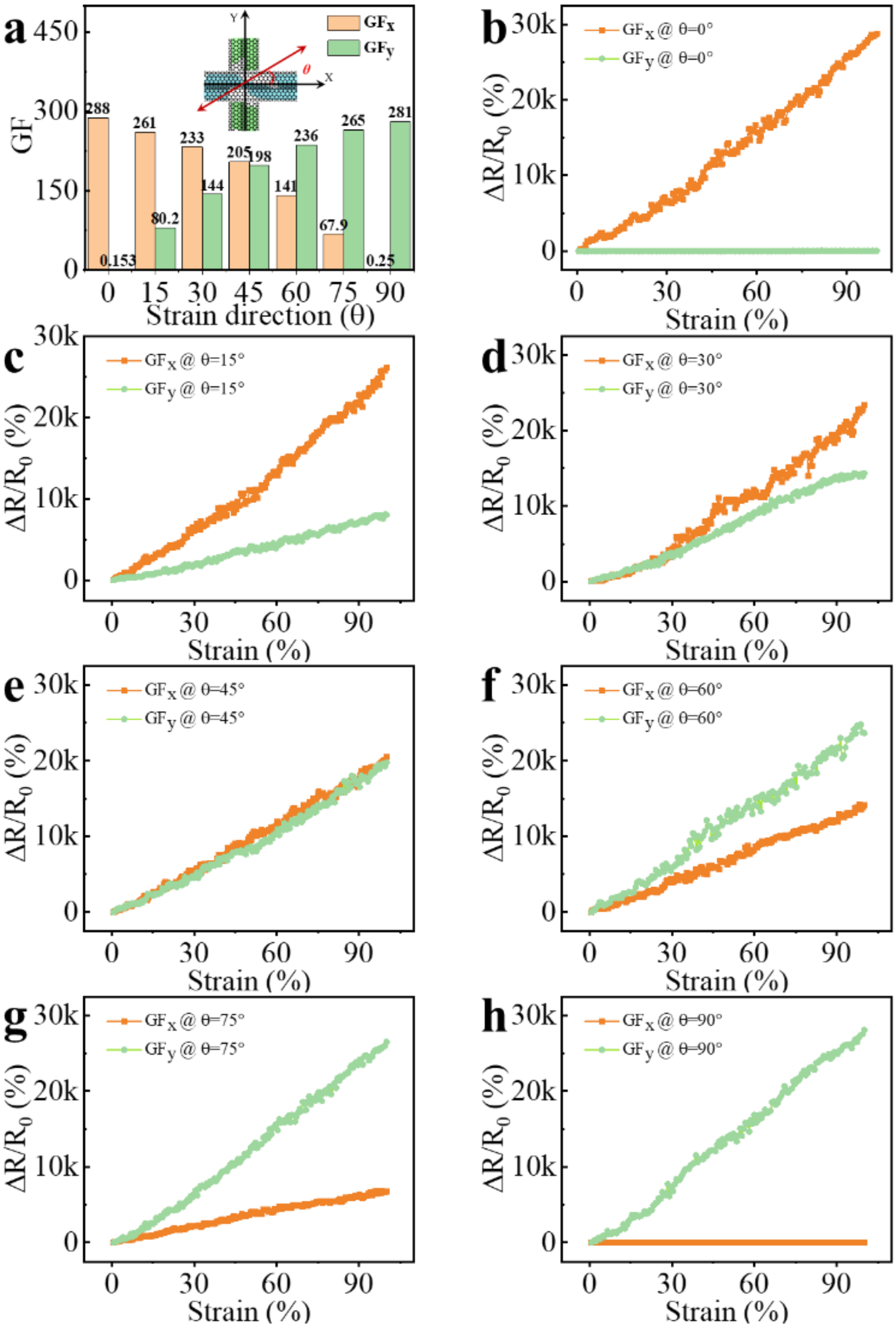

Fig. S24 Directional sensing performance of multidirectional sensors when loaded in different directions. (**a**) GFs measured along two orthogonal axes, GF_x and GF_y, upon application of strains in different directions (θ). The inset schematic shows the conventions of X-, Y-axis, and the loading direction, θ. $\Delta R/R_0$ measured in the X- and Y-axes for θ of (**b**) 0°, (**c**) 15°, (**d**) 30°, (**e**) 45°, (**f**) 60°, (**g**) 75° and (**h**) 90°

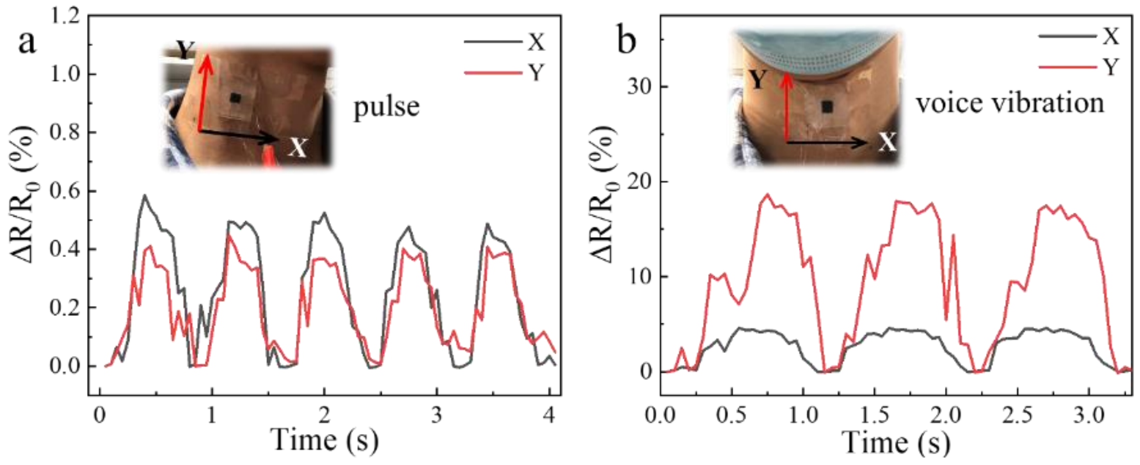

Fig. S25 Detection of human motions involving subtle strains. Relative resistance changes of the multidirectional sensor in response to (**a**) pulse and (**b**) voice vibration

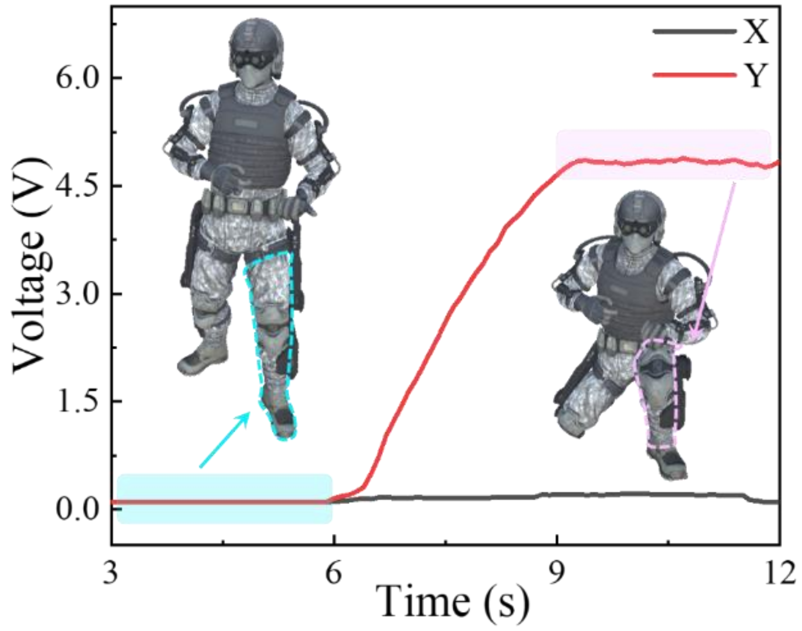

Fig. S26 Animation of deep squat in a 3D character controlled by the multidirectional sensor attached on the knee joint

Table S1 Performance comparison of various multidirectional strain sensors reported in the literature with the current anisotropic bilayer sensor

Structure	Material	Maximum GF	Working range (%)	Selectivity	Linearity	Durability	Refs.
Aligned structure	SWCNTs	59	16%	1.29*	Non-linear	-	[S13]
	MWCNTs	0.04 rad^{-1} (in bending)	200%	0.001*	Linear	15 000 cycles	[S14]
	CNTs	24.3 (0-150%), 437 (150%-260%)	260%	0.54 (0-150%)*	Bi-linear	10,000 cycles	[S15]

	Cellulose nanofiber-CNT hybrid aerogels	1.19	9%	0.026*	Linear	50 cycles	[S16]
	CNT fibers	0.56 (strain: 0-200%) 47 (strain:200%-440%) 64 (strain: 400%-960%)	960%	0.012 (strain: 0-200%) 1.04 (strain:200%-440%) 1.42 (strain: 400%-960%)*	Non-linear	10 000 cycles	[S17]
	CNFs	180	30%	3.84	Non-linear	2500	[S18]
	MWCNTs	2.78	1.5%	0.06*	Bi-linear	-	[S19]
	Vertical graphene	10	10%	0.14*	Linear	21 cycles	[S20]
	Cellulose fibers	10	5%	0.28*	Bi-linear	10 000 cycles	[S21]
Wrinkled structure	CNTs	0.5	600%	0.01*	Linear	5000 cycles	[S10]
	SWCNTs	20	35%	0.48*	Linear	1000 cycles	[S22]
Cracked structure	AgNW fibers	3	30%	0.09*	Linear	5000 cycles	[S23]
	Gold nanowires	10; 1362	79%; 8.8%	0.05*; 30.26*	Bi-linear	1250 cycles	[S24]
	PEDOT:PSS	500* (4%-16%)	16%	11.1	Non-linear	-	[S25]
Microprism-arrayed surface	AgNWs	24.6 (0-130%), 81 (130-150%)	150%	0.34*	Bi-linear	10 000 cycles	[S26]
Stiffness-variant stretchable substrate	AgNWs	20	60%	0.5	Linear	500	[S27]
Molecularly linked thin films	Gold nanoparticles	400	0.8%	8.89*	Non-linear	-	[S28]
Wrinkled and crack-bridging structure	CNT-GO	287.6	100%	6.3	Linear	10000 cycles	This work

*The corresponding selectivity is not specifically reported. They are estimated based on GFs.

Table S2 Fitting values of non-dimensional coefficients of $f(\varepsilon)$ and $g(\varepsilon)$ for sensors made with different area ratios and GO contents

Material parameters	$f(\varepsilon)$				$g(\varepsilon)$				
	A	B	C	D	$k^{\prime}$	$A^{\prime}$	$B^{\prime}$	$C^{\prime}$	$D^{\prime}$
50% area ratio, 25% GO content (Fig. S12a)	144	10	200	2.5	0.75	500	180	6.15	19.2
85% area ratio, 25% GO content (Fig. S12b)	144	15	200	2.2	0.36	500	105	6	15.5

Supplementary Video Clips

Video S1 Strain-dependent morphological change in T-direction of the bilayer sensor

Video S2 Strain-dependent morphological change in L-direction of the bilayer sensor

Video S3 Demonstration of digital character manipulation using the multidirectional sensor

Video S4 Demonstration of sign language recognition using the multidirectional sensor

Supplementary References

[S1] Y. Geng, M.Y. Liu, J. Li, X.M. Shi, J.-K. Kim, Effects of surfactant treatment on mechanical and electrical properties of cnt/epoxy nanocomposites. Compos. Part A **39**(12), 1876-1883 (2008). https://doi.org/10.1016/j.compositesa.2008.09.009

[S2] Q.B. Zheng, W.H. Ip, X.Y. Lin, N. Yousefi, K.K. Yeung et al., Transparent conductive films consisting of ultra large graphene sheets produced by langmuir-blodgett assembly. ACS Nano **5**(7), 6039-6051 (2011). https://doi.org/10.1021/nn2018683

[S3] H. S. Peng, Aligned carbon nanotube/polymer composite films with robust flexibility, high transparency, and excellent conductivity. J. Am. Chem. Soc. **130**(1), 42-43 (2008). https://doi.org/10.1021/ja078267m

[S4] J. A. Swift, Speculations on the molecular structure of eumelanin. Int. J. Cosmetic. Sci. **31**(2), 143-150 (2009). https://doi.org/10.1111/j.1468-2494.2008.00488.x

[S5] D. Chai, Z. Xie, Y. Wang, L. Liu, Y. J. Yum, Molecular dynamics investigation of the adhesion mechanism acting between dopamine and the

surface of dopamine-processed aramid fibers. ACS Appl. Mater. Interfaces **6**(20), 17974-17984 (2014). https://doi.org/10.1021/am504799m

[S6] C. S. Boland, U. Khan, G. Ryan, S. Barwich, R. Charifou et al., Sensitive electromechanical sensors using viscoelastic graphene-polymer nanocomposites. Science **354**(6317), 1257-1260 (2016). https://doi.org/10.1126/science.aag2879

[S7] J. Tersoff. Contact resistance of carbon nanotubes. Appl. Phys. Lett. **74**(15), 2122-2124 (1999). https://doi.org/10.1063/1.123776

[S8] Q. Liu, J. Chen, Y. Li, G. Shi, High-performance strain sensors with fish-scale-like graphene-sensing layers for full-range detection of human motions. ACS Nano **10**(8), 7901-7906 (2016). https://doi.org/10.1021/acsnano.6b03813

[S9] E. Zussman, X. Chen, W. Ding, L. Calabri, D. A. Dikin et al., Mechanical and structural characterization of electrospun pan-derived carbon nanofibers. Carbon **43**(10), 2175-2185 (2005). https://doi.org/10.1016/j.carbon.2005.03.031

[S10] R. Wang, N. Jiang, J. Su, Q. Yin, Y. Zhang et al., A bi-sheath fiber sensor for giant tensile and torsional displacements. Adv. Funct. Mater. **27**(35), 1702134 (2017). https://doi.org/10.1002/adfm.201702134

[S11] J. M. F. Carrasco, E. M. M. Ortega, G. M. Cordeiro, A generalized modified weibull distribution for lifetime modeling. Comput. Stat. Data Anal. **53**(2), 450-462 (2008). https://doi.org/10.1016/j.csda.2008.08.023

[S12] R. Bedi, R. Chandra, Fatigue-life distributions and failure probability for glass-fiber reinforced polymeric composites. Compos. Sci. Technol. **69**(9), 1381-1387 (2009). https://doi.org/10.1016/j.compscitech.2008.09.016

[S13] C. Sui, Y. Yang, R. J. Headrick, Z. Pan, J. Wu et al., Directional sensing based on flexible aligned carbon nanotube film nanocomposites. Nanoscale **10**(31), 14938-14946 (2018). https://doi.org/10.1039/c8nr02137f

[S14] H. Zhu, X. Wang, J. Liang, H. Lv, H. Tong et al., Versatile electronic skins for motion detection of joints enabled by aligned few-walled carbon nanotubes in flexible polymer composites. Adv. Funct. Mater. **27**(21), 1606604 (2017). https://doi.org/10.1002/adfm.201606604

[S15] L. Ma, W. Yang, Y. Wang, H. Chen, Y. Xing et al., Multi-dimensional strain sensor based on carbon nanotube film with aligned conductive networks. Compos. Sci. Technol. **165**, 190-197 (2018). https://doi.org/10.1016/j.compscitech.2018.06.030

[S16] C. Wang, Z. Z. Pan, W. Lv, B. Liu, J. Wei et al., A directional strain sensor based on anisotropic microhoneycomb cellulose nanofiber-carbon nanotube hybrid aerogels prepared by unidirectional freeze drying. Small **15**(14), e1805363 (2019). https://doi.org/10.1002/smll.201805363

[S17] S. Ryu, P. Lee, J. B. Chou, R. Z. Xu, R. Zhao et al., Extremely elastic wearable carbon nanotube fiber strain sensor for monitoring of human motion. ACS Nano **9**(6), 5929-5936 (2015). https://doi.org/10.1021/acsnano.5b00599

[S18] J.-H. Lee, J. Kim, D. Liu, F. Guo, X. Shen et al., Highly aligned, anisotropic carbon nanofiber films for multidirectional strain sensors with exceptional selectivity. Adv. Funct. Mater. **29**(29), 1901623 (2019). https://doi.org/10.1002/adfm.201901623

[S19] A. I. Oliva-Avilés, F. Avilés, V. Sosa, Electrical and piezoresistive properties of multi-walled carbon nanotube/polymer composite films aligned by an electric field. Carbon **49**(9), 2989-2997 (2011). https://doi.org/10.1016/j.carbon.2011.03.017

[S20] S. Huang, G. He, C. Yang, J. Wu, C. Guo et al., Stretchable strain vector sensor based on parallelly aligned vertical graphene. ACS Appl. Mater. Interfaces **11**(1), 1294-1302 (2019). https://doi.org/10.1021/acsami.8b18210

[S21] S. Chen, Y. Song, D. Ding, Z. Ling, F. Xu, Flexible and anisotropic strain sensor based on carbonized crepe paper with aligned cellulose fibers. Adv. Funct. Mater. **28**(42), 1802547 (2018). https://doi.org/10.1002/adfm.201802547

[S22] K. K. Kim, S. Hong, H. M. Cho, J. Lee, Y. D. Suh et al., Highly sensitive and stretchable multidimensional strain sensor with prestrained anisotropic metal nanowire percolation networks. Nano Lett. **15**(8), 5240-5247 (2015). https://doi.org/10.1021/acs.nanolett.5b01505

[S23] Y. Cheng, R. Wang, H. Zhai, J. Sun, Stretchable electronic skin based on silver nanowire composite fiber electrodes for sensing pressure, proximity, and multidirectional strain. Nanoscale **9**(11), 3834-3842 (2017). https://doi.org/10.1039/c7nr00121e

[S24] S. Gong, L. W. Yap, B. Zhu, Q. Zhai, Y. Liu et al., Local crack-programmed gold nanowire electronic skin tattoos for in-plane multisensor integration. Adv. Mater. **31**(41), 1903789 (2019). https://doi.org/10.1002/adma.201903789

[S25] B. Sarkar, D. K. Satapathy, M. Jaiswal, Wrinkle and crack-dependent charge transport in a uniaxially strained conducting polymer film on a flexible substrate. Soft Matter. **13**(32), 5437-5444 (2017). https://doi.org/10.1039/c7sm00972k

[S26] K. H. Kim, N. S. Jang, S. H. Ha, J. H. Cho, J. M. Kim, Highly sensitive and stretchable resistive strain sensors based on microstructured metal nanowire/elastomer composite films. Small **14**(14), e1704232 (2018). https://doi.org/10.1002/smll.201704232

[S27] S. H. Ha, S. H. Ha, M. B. Jeon, J. H. Cho, J. M. Kim, Highly sensitive and selective multidimensional resistive strain sensors based on a stiffness-variant stretchable substrate. Nanoscale **10**(11), 5105-5113 (2018). https://doi.org/10.1039/c7nr08118a

[S28] W. Zhao, J. Luo, S. Shan, J. P. Lombardi, Y. Xu et al., Nanoparticle-structured highly sensitive and anisotropic gauge sensors. Small **11**(35), 4509-4516 (2015). https://doi.org/10.1002/smll.201500768
